# Imaging lymphatic function and inflammation response through hypoxia via endogenous biomarker

**DOI:** 10.1117/1.JBO.31.1.016003

**Published:** 2025-12-29

**Authors:** Marien I. Ochoa, Xu Cao, Matthew S. Reed, Eduard Matkovic, Weifeng Zeng, Samuel O. Poore, Brian W. Pogue

**Affiliations:** a University of Wisconsin-Madison, Department of Medical Physics, Madison, Wisconsin, United States; b Thayer School of Engineering at Dartmouth, Hanover, New Hampshire, United States; c University of Wisconsin-Madison, Department of Pathology, Madison, Wisconsin, United States; d University of Wisconsin School of Medicine and Public Health, Department of Surgery, Madison, Wisconsin, United States

**Keywords:** fluorescence imaging, lymphatic function, oxygen sensing, sentinel node detection, medical imaging

## Abstract

**Significance:**

The lymphatic system, crucial for immune function and fluid balance, is difficult to study due to its nearly invisible channels and passive movements. Despite its importance, real-time, noninvasive lymphatic imaging is limited and often relies on exogenous agents. A largely overlooked feature of the lymphatic system is its natural hypoxia, arising because lymphatic vessels and nodes are distant from oxygen-rich blood and serve as a waste reservoir and from amplifying factors such as inflammation. This hypoxia has been linked to lymphangiogenesis and cancer metastasis.

**Aim:**

We introduce a method for real-time macroscopic imaging of lymphatic function and response to inflammation, through the intrinsic hypoxia transients that occur in lymphatic structures.

**Approach:**

The naturally hypoxic environment of the lymphatic system was imaged *in vivo* in mice through the delayed fluorescence (DF) of metabolized endogenous protoporphyrin IX (PpIX), induced by 5-aminolevulinic acid. PpIX localizes to inflamed regions; when inflammation is cleared by lymphatics, the low oxygen conditions cause the DF signal to be amplified. DF imaging and lymphatic kinetics were characterized in wound, inflammation, and pancreatic tumor models. High-intensity popliteal and sentinel nodes, wounds, and tumors were excised for immunohistochemistry (IHC) and hematoxylin and eosin analysis.

**Results:**

Lymphatic pumping frequency changed with increasing wound severity, and hypoxia appeared in sentinel nodes near tumors. Cyclical pumping occurred at edema sites and in wound and tumor-adjacent nodes. Uninjured anesthetized mice showed little contrast, whereas awake mice exhibited hypoxia localized to lymph nodes. Microscopy and IHC confirmed PpIX and hypoxia presence in nodes, tumors, and wounds, localized to macrophages and T cells.

**Conclusions:**

Unlike injection-based regional lymph node mapping, DF hypoxia imaging appears to provide a natural whole-body contrast mechanism, highlighting its potential for visualizing lymphatic function and associated hypoxia dynamics. This original documentation of lymphatic hypoxia has potential applications in surgical guidance, tracking of metastatic tumors, and immune response tracking.

## 1 Introduction

To date, the lymphatic network—one of the major circulatory systems in the body—has not been nearly as well studied as other circulatory or transport processes.^1^ This is mainly due to its complexity, passive, and nearly invisible nature—as lymphatic channels are not easily observed by human vision in comparison to cardiovascular structures.^2^ In addition, unlike the cardiovascular system, movements are largely passive and not coordinated through larger structures (e.g., cardiac pumping).^3^^,^^4^ However, the lymphatic system is a crucial part of the body’s immune response due to its role in the transport of white blood cells, providing an isolated system to protect the organism against invaders and infections. Hence, understanding how the lymphatic system responds to treatment (e.g., immunotherapy) has gained attention.^5^ Another essential function of the lymphatic system is the prevention of edema through the regulation of fluids in conjunction with the cardiovascular system.^1^ Hence, the capabilities of the lymphatic system to properly regulate immune cell production and function, fat absorption, and overall fluid balance play a vital role in protecting the body against infections and supporting overall health. However, unlike the circulatory system, which is highly regulated to match oxygen supply, the lymphatic system is regulated by multiple decentralized local mechanisms, such as osmotic pressure, the intrinsic contractions of lymphangions, external forces, and shear stress.^6^^,^^7^ We hypothesize here that it can therefore undergo periods of deep hypoxia when overloaded in imbalanced homeostatic conditions such as tissue inflammation or cancer growth. Imaging the function of the lymphatics is challenging because lymph capillaries are small with visually clear fluid and slow flow rates. Several critically important studies have shown active pumping mechanisms of lymphatics with regional injection of contrast agents;^8^ however, this current study extends this to examine when lymph flow becomes hypoxic based on metabolic overload of the oxygen supply.

**Fig. 1:**
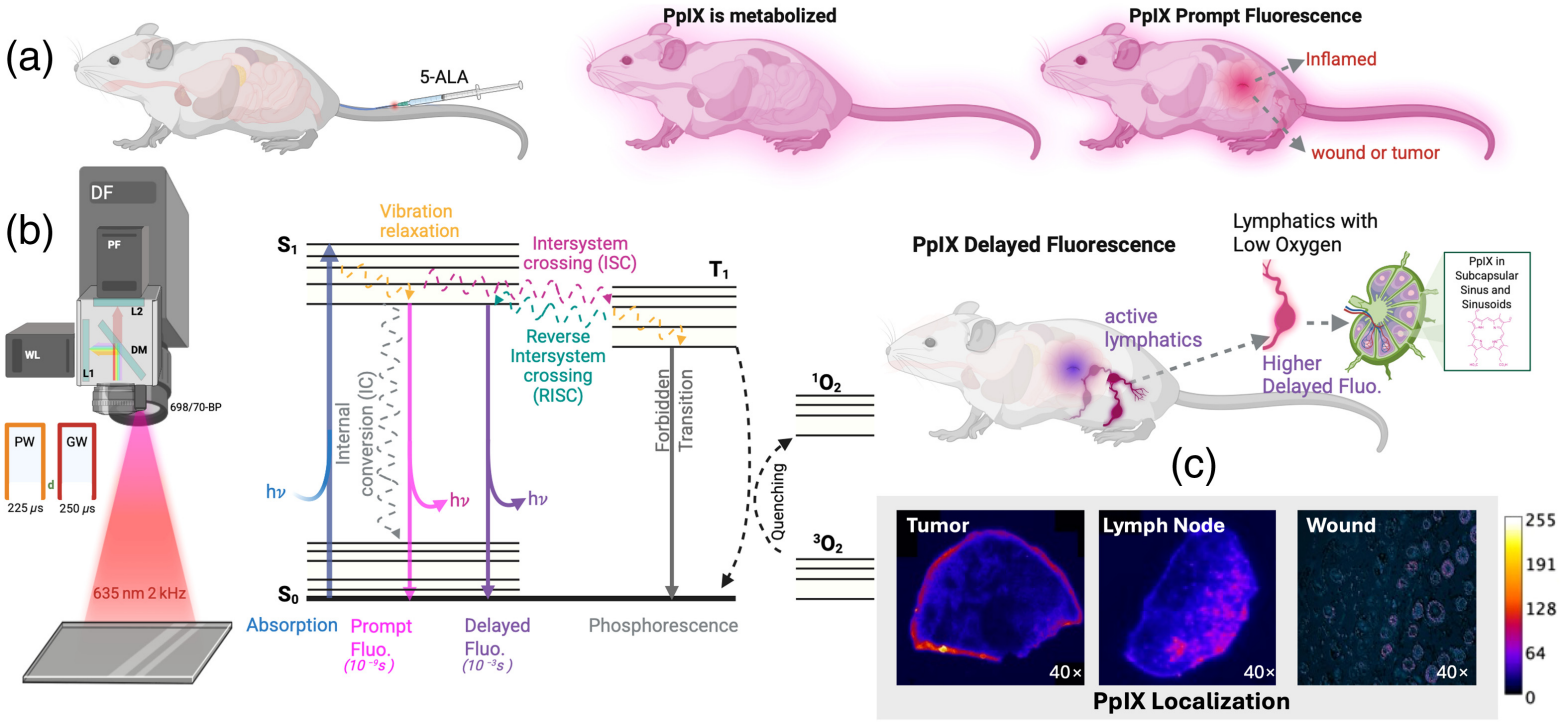
Illustration principle of hypoxia imaging in lymphatics with PpIX as a marker for intracellular hypoxia. (a) Depiction of the contrast mechanism through PpIX DF. (b) Depiction of a designed optical instrument for DF lymphatic hypoxia imaging. (c) Depiction of the presence of PpIX, for example, tumors, lymph nodes, and wounded tissue, as imaged in microscopy for histological samples. PW-pulse width, GW-gate width, and d (delay) parameters represented in panel (b).

At present, imaging of lymphatic structure and flow is achieved with local exogenous contrast injections. Several imaging techniques such as ultrasound imaging,^9^ bio-impedance imaging,^8^ magnetic resonance imaging,^10^ lymphoscintigraphy,^11^ lymphangiography,^12^ dynamic contrast-enhanced magnetic resonance lymphangiography,^13^ and near-infrared/indocyanine green lymphography^14^ are used. However, the features of lymphatic function that have been documented are still quite limited in terms of metabolism and contents of the lymph fluids.^8^ An important and unique feature of the lymphatic system is the dynamic response due to damage that can lead to cellular overload and hypoxia,^15^^,^^16^ although tools to study this are limited. Hypoxia in lymphatics can exist naturally and results from lymph vessels and nodes being frequently situated in remote areas far from oxygen-carrying blood vessels.^16^^,^^17^ Hypoxia might also be expected because lymphatics are a less actively regulated system for lymph fluid carrying molecules based on a complex milieux of interstitial pressures, convection, and cellular uptake. During times of high metabolic demand for oxygen in the body, it seems likely that extracellular fluid can be transiently depleted of oxygen. The microenvironment where lymphatic vessels reside inherently has a lower oxygen concentration than the areas immediately surrounding blood vessels, which serves as its baseline state. This inherent vulnerability is dramatically amplified under pathological states such as tumors and inflammation, where high interstitial fluid pressure compresses lymphatic vessels and blood vessels to further increase the resistance for oxygen diffusion while local oxygen consumption increases due to higher immune activities. Both the baseline state and amplifying factors can amplify lymph hypoxia conditions. Previous studies have reported on the role of hypoxic conditions in the lymphatic system in promoting lymphangiogenesis and metastatic spread through the lymphatic channels.^15^^–^^17^ To date, imaging of oxygen content in the lymphatic network has been mainly explored in the microscopic regime for *ex vivo* tissues with exogenous labels.^18^ The work presented in this study establishes the use of endogenous protoporphyrin IX (PpIX) delayed fluorescence (DF) optical imaging as a method to macroscopically image hypoxia dynamics in lymphatics *in vivo* and in real time. As depicted in Fig. 1, endogenous PpIX production can be enhanced by precursor 5-aminolevulinic acid (5-ALA), which is already FDA approved for human use.^19^ PpIX possesses both a prompt fluorescence (PF) and a DF component. The intensity of the PF component is representative of PpIX concentration. On the other hand, the DF component results from the triplet state of the molecule, which is quenched by oxygen.^20^^,^^21^ In hypoxic conditions, the delayed fluorescence lifetime increases to the millisecond regime, and hence, the DF signal can be readily recovered by time-gating and collecting after each excitation pulse, whereas PF signals can be recovered by imaging during the excitation pulse.^22^ A summary of the setup is depicted in Fig. 1.

To our knowledge, this work explores for the first time the response of hypoxia in lymphatics to wound and tumor models, using endogenous PpIX as an oxygen sensor and the natural hypoxic state of lymphatics to create a contrast mechanism for imaging. As briefly depicted in Fig. 1, this study will microscopically confirm the presence of PpIX in lymphatic, tumor, and wound murine models while also providing immunohistochemistry (IHC) analysis to better understand the nature of the PpIX signal. At the macroscopic level, the localization of lymphatics through PpIX DF optical imaging will be correlated to lymphatic vessels and nodes labelled through ICG^23^^,^^24^ for uninjured mice. Lymphatic function will be subsequently evaluated for mice *in vivo* through induced moderate and severe wound injuries, with uninjured mice serving as controls. Real-time PpIX DF imaging will be also performed for mice in movement. Finally, PpIX DF imaging will be used to dynamically (i.e., in real time) image the lymphatic response to pancreatic adenocarcinoma (AsPC1) tumors *in vivo*.

## 2 Results

### 2.1 Macroscopic PpIX DF Hypoxia Imaging Dynamics in Relation to Vital Signs

**Fig. 2:**
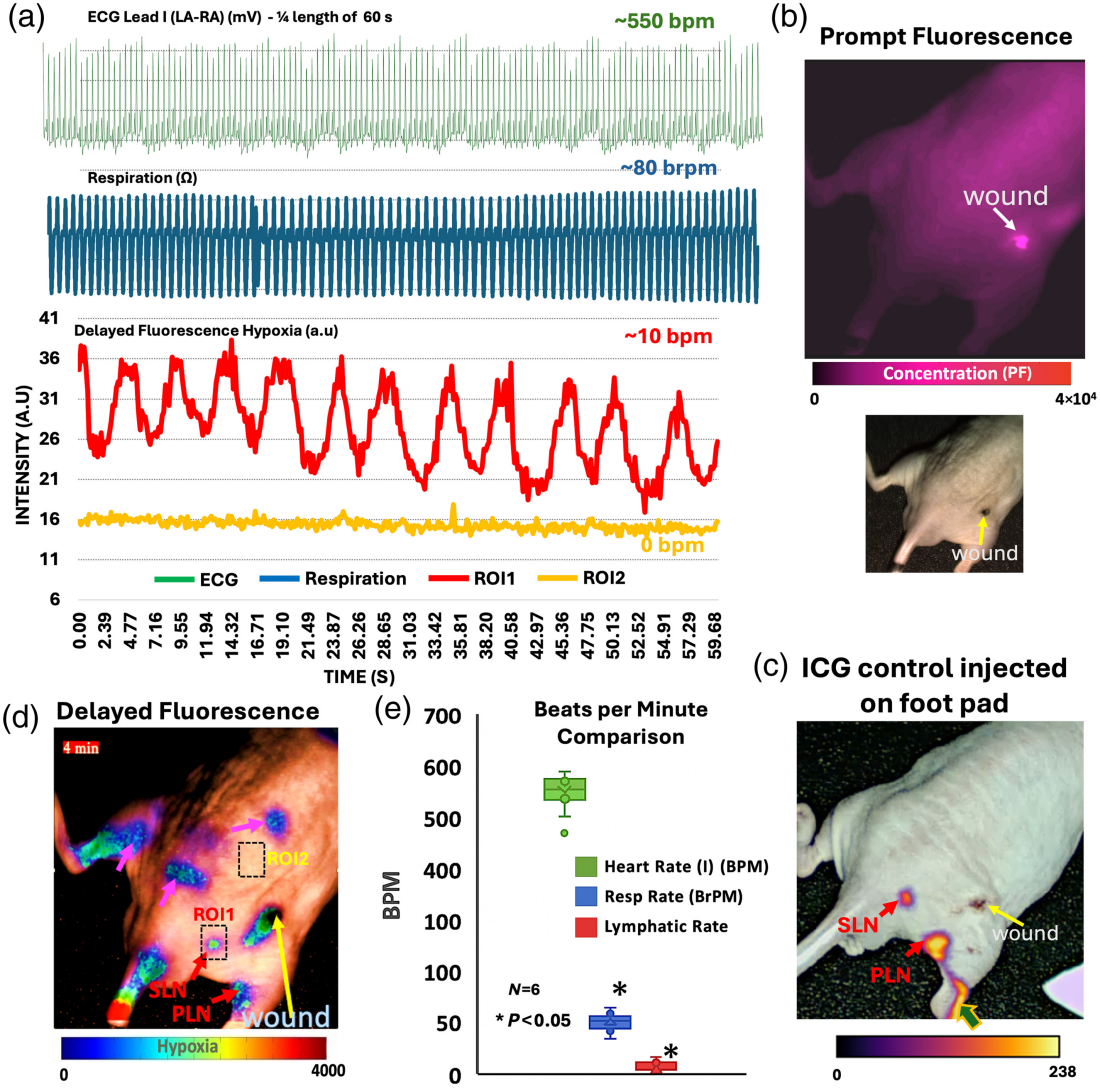
Example lymph response as recorded by PpIX DF hypoxia imaging for a mouse with a moderate level of wound injury. (a) Example and comparison of a recorded signal for heart rate, respiration rate, and PLN lymph node for a mouse displayed in panel (d). (b) PpIX prompt fluorescence (PF) channel representative of PpIX concentration. (c) ICG injected in the footpad to track neighboring nodes and verify the location. (d) PpIX DF hypoxia frame at 4 min with lymph nodes that correlate to ICG (red arrows) and additional areas identified through PpIX DF hypoxia imaging (pink arrows). (e) Quantification of beats/pulsations per minute (BPM) of PLN and SLN nodes in comparison to BPM recorded for respiration and heart rate. Real-time kinetics and movements can be appreciated in Videos 1(a)–1(c). Video 1(c), which represents PpIX PF, displays no localization to lymphatics in comparison to PpIX DF and hypoxia in Video 1(a) (Video 1, MP4, 14.4 MB [URL: https://doi.org/10.1117/1.JBO.31.1.016003.s1]).

To confirm that the detected DF hypoxia signals corresponded to lymphatics, simultaneous ICG lymph node imaging was used to visualize the location of the same lymph nodes closer to the wound area, as described in Sec. 5. In addition, heart rate and respiration rate were monitored to better understand if pulsations happened at the same frequencies. The use of hairless male nude mice was preferred to reduce hair absorption and scattering effects. Figure 2 displays these results, where Fig. 2(b) shows the PF signal of PpIX representative of PpIX distribution and concentration on the intact mouse. Across mice, it was observed that PpIX PF was higher at the wound site with fluorescence also present across the mouse skin. ICG injected in the right foot pad was used to localize both popliteal (PLN) and sciatic (SLN) lymph nodes, as shown in Fig. 2(d), and to validate if PpIX also reported those same lymph nodes. With PpIX-based hypoxia imaging, both PLN and SLN nodes displayed draining activity, as shown in Fig. 2(d), where frames were selected from the real-time sequence at 4 min and for dynamic display purposes at 1, 2, and 3 min after imaging started in Fig. S1 in the Supplementary Material.

Figure 2(d) displays minute 4 of the real-time imaging sequence; however, Supplementary Video S1 displays the dynamic temporal nature of the signal. In this example, the nodes that correlated to ICG are indicated with red arrows, and nodes and structures that were not located by ICG but only by PpIX DF imaging are indicated in a different color. Notably, DF PpIX imaging displayed more areas of activity across the mouse than ICG imaging. ICG injection on the footpad only reached the SLN and PLN nodes as previously reported.^24^^–^^26^ Pulsation effects were not observed in the PLN or SLN node through ICG but were observed through PpIX DF imaging. Quantification of these pulsations is displayed in Fig. 2(e) for the group of mice. An example of the pulsation sequence of PpIX DF intensity from the PLN region is shown in Fig. 2(a) for an interval of 1 min versus a control region. PpIX DF imaging was compared with measurements acquired with a vital sign monitor, where heart rate and respiration signals were recorded over a consecutive period of 12 min. Lymphatic signals recovered through DF imaging of PpIX pulsed at a slower rate compared with respiration and heart rate signals at an average of ∼10 pulses per minute (bpm), as quantified in Fig. 2(e). An example of the average oscillation pattern for the lymph node region versus normal skin is presented in Fig. 2(a), and beats per minute (BPM) are annotated. It can be observed that hypoxia pulsations on the skin area are negligible and different from those observed in lymph node areas. As there is PpIX on the skin, as shown in the prompt fluorescence channel, which is representative of PpIX concentration, the lack of skin oscillations should be mostly due to the lack of skin hypoxia and not to the lack of PpIX. In addition, the oscillation pattern for a control *in vitro* vial is exemplified in Fig. 7(e). The vial lacks oscillations as expected. These controls rule out aliasing as, if present, it should be observed across the whole sensor. Hence, it has been validated that the signal is inherent to biological changes within the mouse rather than system artifacts.

### 2.2 PpIX Microscopic Localization in Tumors, Lymph Nodes, and Wound Regions

**Fig. 3:**
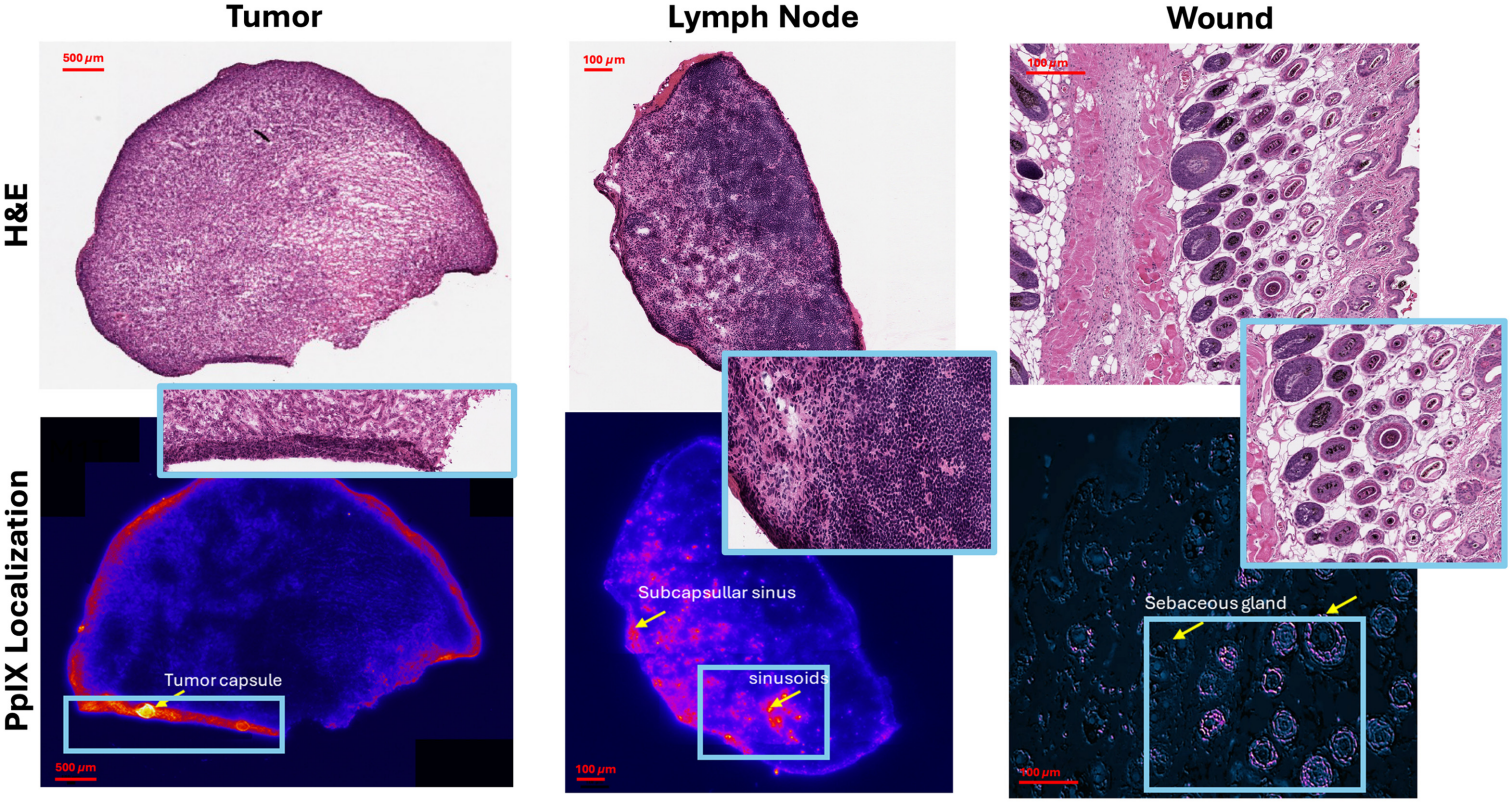
Verification of PpIX in tumor, lymph nodes, and wounds in mice at 1 h post 5-ALA injection, with PpIX fluorescence imaging at 40× magnitude for the whole region of interest. Localization in the tumor is around the capsule, where active capillaries are. Localization in the lymph node is in the subcapsular sinus areas, and localization in the skin wound area is in the sebaceous glands.

The lymph nodes and areas that macroscopically displayed high DF hypoxia of PpIX, namely, tumor, lymph node, and wound regions, were excised and embedded in O.C.T to preserve the endogenous PpIX signals. Example results for tumor, lymph nodes, and wound models are displayed in Fig. 3. Based on PpIX microscopy imaging (20×) and histology analysis, the localization of PpIX in tumors is constrained to a connective tissue capsule surrounding the tumor region. On the other hand, the localization of PpIX fluorescence in lymph nodes came from the subcapsular sinus and medullary sinusoids. In subcapsular sinuses, macrophages form the first cell layer to drain lymph fluid, capturing and retaining pathogens from infiltrating other node regions.^27^ The medullary sinuses are known to gather lymph from the cortical and trabecular sinuses and are composed of histiocytes and reticular cells.^28^ For wounds, the presence of PpIX fluorescence was dominant in hair follicle areas and sebaceous glands with less intensity than PpIX observed in lymph nodes and tumors, as exemplified in Fig. 3. These results highlight the accumulation of PpIX in regions where PpIX-based hypoxia imaging highlighted activity. Hence, both PpIX microscopy and macroscopic results display PpIX accumulation in tumors, lymph nodes, and wounds. As multiple biological structures were present, equivalent tissues were also analyzed through IHC (further below) to further understand which structures expressed PpIX-based hypoxia and matched PpIX microscopy localization.

### 2.3 Factors behind Microscopic Localization and Hypoxia Validation of Tissues Through IHC

**Fig. 4:**
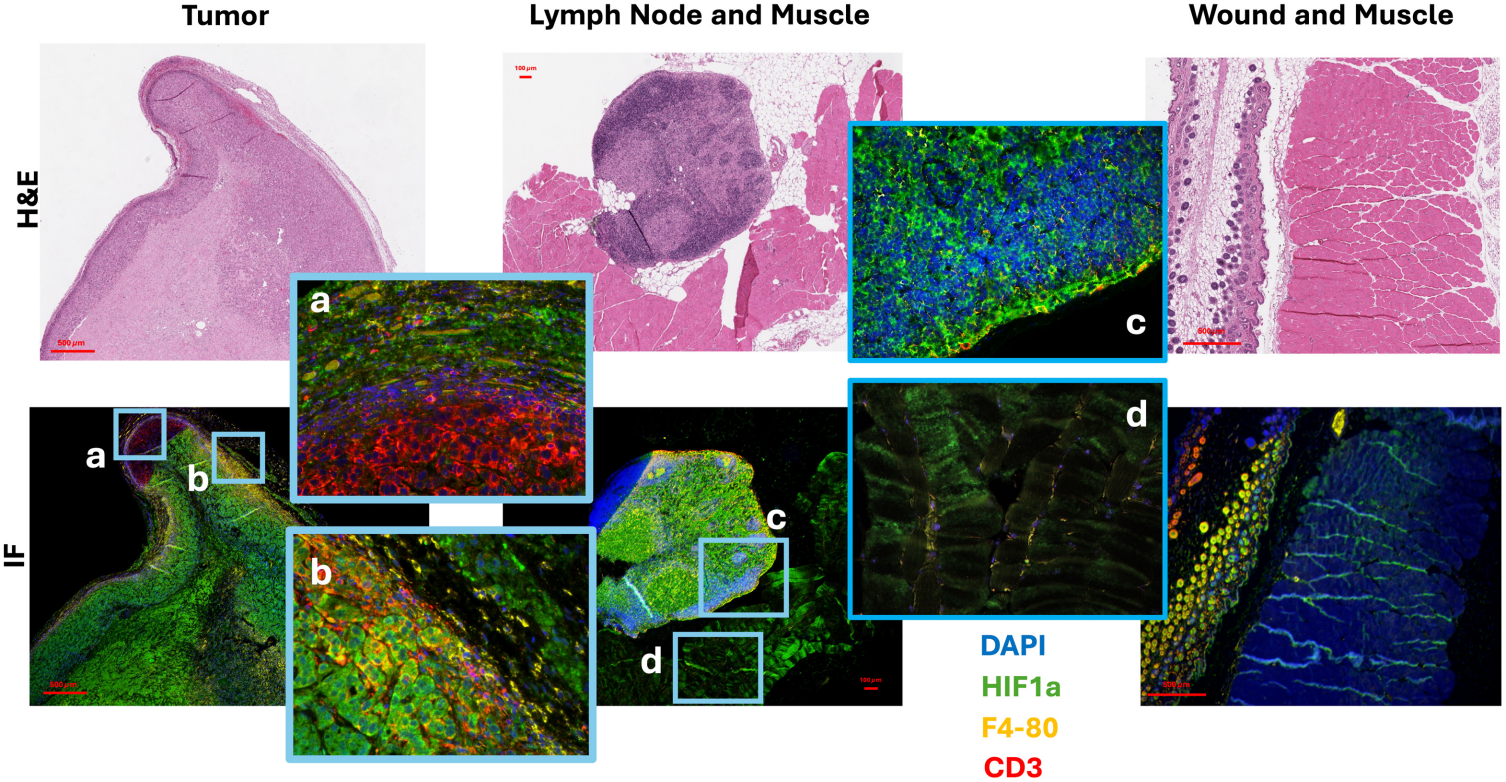
Verification of hypoxia in tumor, lymph nodes, and normal tissue at 1 h post 5-ALA injection, with IHC staining and multispectral fluorescence imaging. Example images at 40× magnification for tumor, lymph node, wound, and muscle tissue. Corresponding H&E images are provided in the first row. In addition, composite images of IHC stains are displayed with zooming in respective areas a, b, c, and d. Composite images are color-coded to represent DAPI in blue, HIF-1α in green, F4-80 in yellow, and CD3 in red.

Through IHC staining and multispectral microscopy, equivalent tissues to those imaged with PpIX microscopy were scanned for hypoxia-inducible factor 1 alpha (HIF-1α) (hypoxia marker), F4/80 (macrophage marker), CD3 (T cell marker), and DAPI. Example results are shown in Fig. 4 for tumor, lymph node, wound, and muscle tissues. Additional datasets are displayed in Fig. S2 in the Supplementary Material for an example set of lymph nodes from different mice, in Fig. S3 in the Supplementary Material for a set of AsPC1 tumors from different mice, and in Fig. S4 in the Supplementary Material for a set of wounds. Supplementary figures display the unmixed antibody stains and their location as well as respective H&E images. Figures S2 and Fig. 4 (c and d) confirm the presence of higher HIF-1α in lymph nodes compared to normal muscle tissue. HIF-1α is a transcription factor and hypoxia response element that regulates gene expression in low oxygen conditions. Hence, it is expected that it regulates essential processes such as metabolism and angiogenesis.^29^

The results indicate high levels of HIF-1α in regions that correlate to high PpIX-delayed fluorescence intensity when macroscopically imaged, namely, tumors, lymph nodes, and wounds. Based on IHC results for lymph nodes, the regions where PpIX was located in PpIX microscopy imaging (subcapsular sinus and medullary sinusoids) correlate to the presence of not only hypoxia factor HIF-1α but also both macrophages (F4/80) and T cells (CD3), suggesting the possible intervention of both immune cells in the process of PpIX clearance and the dynamic signals captured through the PpIX delayed fluorescence macroscopic imager for lymph nodes. The lymph node pulsation mechanism exemplified in Fig. 2(b) will then be hypothesized to have its origin in the presence of PpIX, a hypoxia factor in the lymph nodes, and the clearance of PpIX from lymph nodes through macrophages and T cells. In addition to nodes, IHC staining for tumors also reveals the presence of hypoxia factor in the connective tissue capsule surrounding the tumor and additionally correlates to the presence of macrophages and T cells. For wounded tissue, PpIX presence was dominant in hair follicles and sebaceous glands. Furthermore, for wounds, these areas were correlated to the presence of macrophages and T cell infiltration.

### 2.4 PpIX Localization and Hypoxia *In Vivo* Validation with Local Alternative Oxygen Probe

**Fig. 5:**
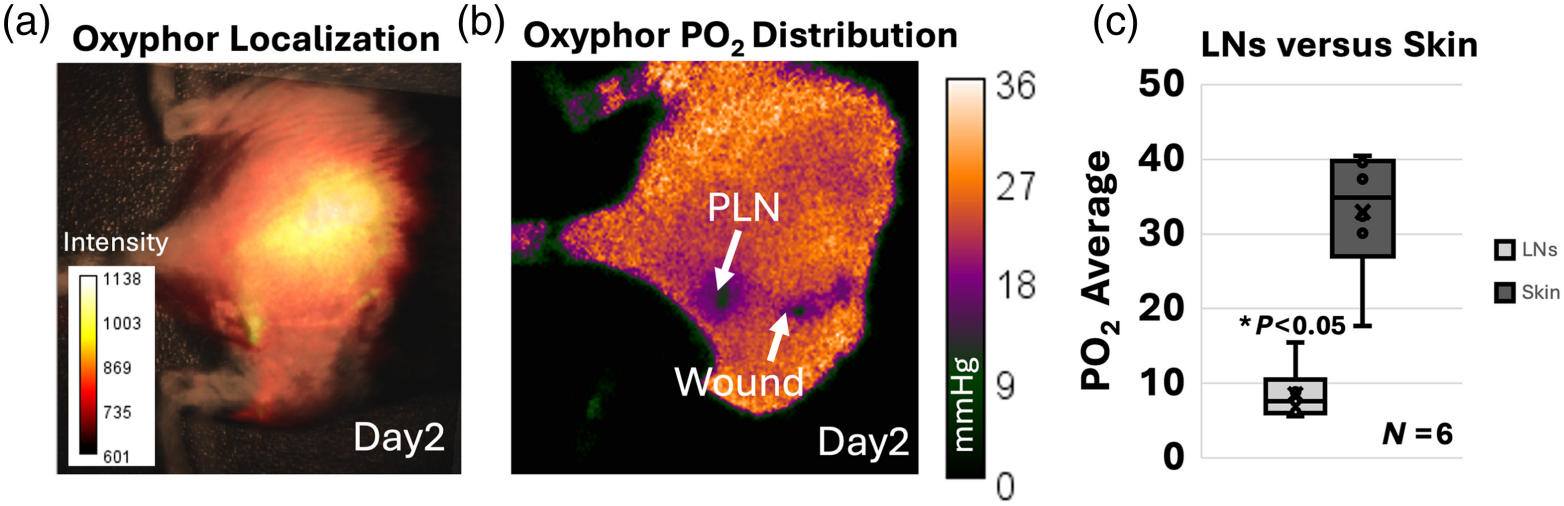
Oxygen tension (${\mathrm{PO}}_{2}$) as quantified through PDG4 injectable oxygen sensing probe (Oxyphor) in lymph node areas, wound areas, and normal tissue. (a) Localization of PdG4 on day 2 post hind-paw injection. (b) Corresponding PO2 distribution as mapped with macroscopic-based imaging of PdG4. (c) Quantification of ${\mathrm{PO}}_{2}$ average for lymph nodes (LNs) versus skin for N=6.

The results for independent oxygen sensing to validate the studies were obtained at day 1 and day 2 post PdG4 Oxyphor injection in the right hind leg of mice. On day 1 (same day as injection) across the mice dataset (N=5) and after massage of the hind paw following injection, the probe entered the PLN and SLN nodes and their connected lymphatic vessels, as displayed in Fig. S5 in the Supplementary Material. Measurements were then performed on day 2 post administration after the probe had distributed to neighboring areas, providing some control data from other regions different than the nodes [Fig. 5(a)]. Results from the OxyLED reader are summarized in Fig. 5(c), indicating an average ${\mathrm{pO}}_{2}$ of 10 mmHg for the PLN areas. In comparison, results for normal tissue on the medial dorsal region had an average ${\mathrm{pO}}_{2}$ of 38 mmHg. The wound area was also shown as low in oxygen, with ${\mathrm{pO}}_{2}$ values ranging from 18 to 20 mmHg. The results highlighted lower oxygen values in the PLN for days 1 and 2 post-injection and higher oxygen values for normal dorsal regions. An example of a ${\mathrm{pO}}_{2}$ distribution map as measured with macroscopic imaging of PdG4 Oxyphor is shown in Fig. 5(b) for day 2. Macroscopic imaging and recorded ${\mathrm{pO}}_{2}$ values are in accordance with values measured with the OxyLED fiber-based probe for PLN regions and medial dorsal control regions. In addition to the IHC results, ${\mathrm{pO}}_{2}$ tension measurements also verify the presence of low oxygen in lymph nodes, specifically for PLNs *in vivo* in comparison to normal tissue, as well as the presence of low oxygen in wound regions as observed through IHC HIF-1α staining. Oxyphor contrast successfully confirmed the relative differences in PLN nodes in this region and estimated ${\mathrm{pO}}_{2}$ values with both macroscopic imaging and fiber-probe methods.

### 2.5 Movement Enhances Response and Visualization of Lymph Nodes

**Fig. 6:**
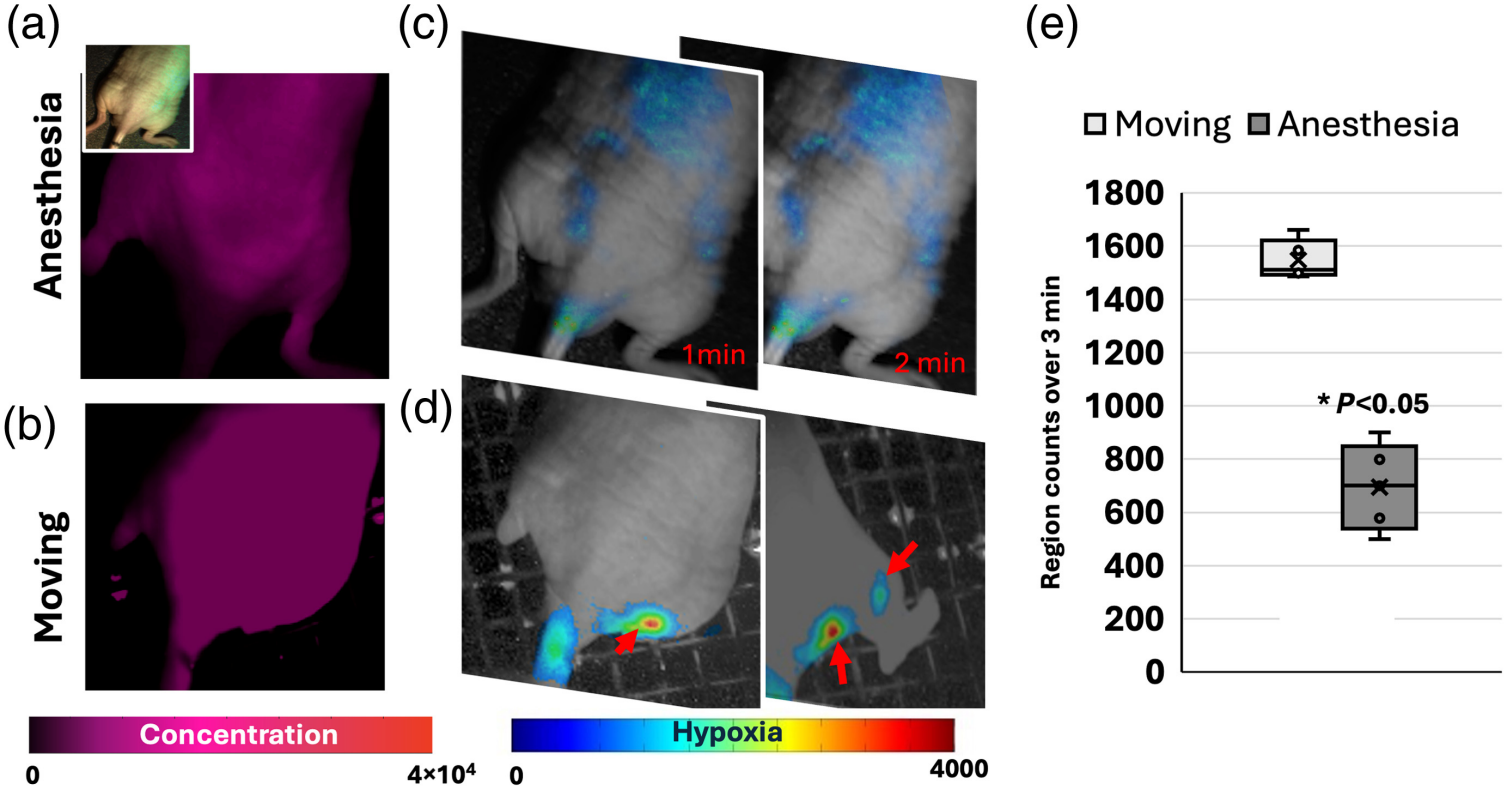
Lymphatic response through PpIX DF imaging for a noninjured mouse with and without anesthesia (awake and moving). (a) White-light and PpIX prompt fluorescence (PF) images. (b) PF for an example frame at 2 min of the mouse without anesthesia. (c) PpIX DF hypoxia with example frames over time. (d) PpIX DF hypoxia for moving the mouse with red arrows pointing at identified lymph nodes. (e) Regions count over 3 min for N=4 for moving versus anesthetized mice. Hypoxia images were overlaid on a gray-scale white-light image for visualization purposes. Real-time kinetics and movements can be appreciated in Videos 2(a) and 2(b) (Video 2, MP4, 1.47 MB [URL: https://doi.org/10.1117/1.JBO.31.1.016003.s2]).

Due to the passive movements expected from the lymphatic structures^3^^,^^4^ and their predicted faster function under movement, real-time PpIX DF imaging was performed on uninjured mice in movement as described in Sec. 5. The results are shown in Fig. 6 for a mouse under anesthesia and subsequently awake and ambulating. The results as exemplified in Fig. 6(a) showed PpIX PF distributed throughout the mouse when mice were anesthetized and when mice were awake and moving. Unlike wounded mice models, PpIX did not accumulate more in a specific area (e.g., wound) as per PF images in Figs. 6(a) and 6(b). When the mouse was ambulated [Figs. 6(c) and 6(d)], signals originating from the PLN and SLN lymph nodes in areas that were close to the tail and flank of the hind legs were observed on the DF hypoxia channel. Unlike mice under anesthesia, the hypoxia signal intensity was localized to the PLN and SLN nodes when mice were moving. Due to the complexity of quantifying lymphatic pulsation effects for moving mice, only intensity counts were quantified for frames where mice remained calm, and intensities were averaged at the hind leg and back regions of the mouse over a fixed period. This was quantified for the group (N=4) by averaging the overall intensity counts on the flanks of the hind legs of the mouse over a span of 3 min. Quantification is shown in Fig. 6(e), where PpIX DF intensity counts were higher at the flank and hind-leg regions when the mouse was in movement. Of note, Fig. 2(c) illustrates that when the intensity was lower due to anesthetic effects, dynamic signals were also captured from the chest region.

### 2.6 Lymphatic Response in the Presence of Tissue Injury Swelling

**Fig. 7:**
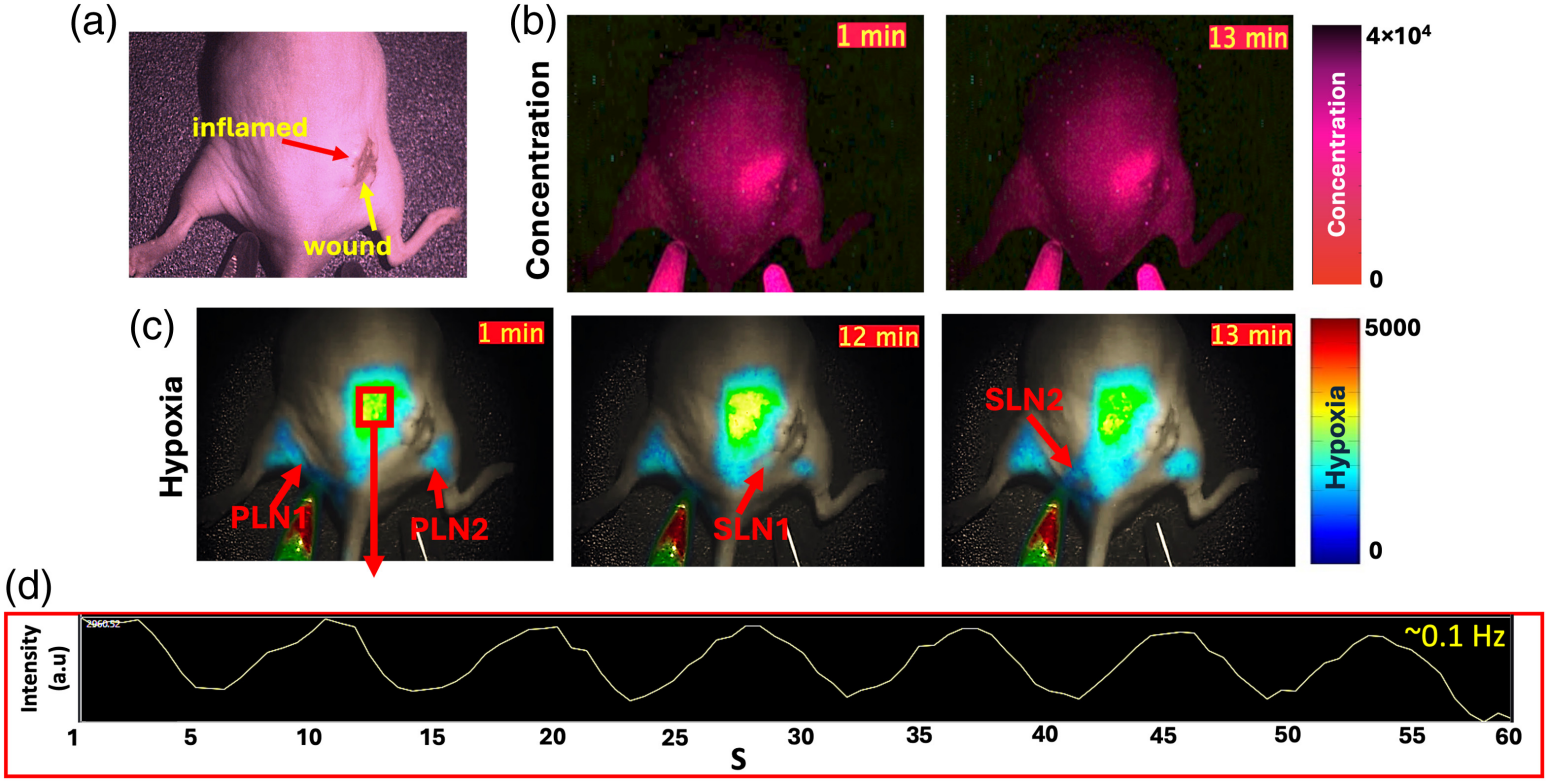
Example of lymphatic response as recorded with PpIX DF imaging for a severely injured mouse model. (a) Visible light image of a mouse model. (b) PpIX prompt fluorescence (PF) channel representative of PpIX concentration at 1 and 13 min after imaging started. (c) PpIX delayed fluorescence (DF) hypoxia channel with example frames over time and highlighting PLN and SLN nodes in intermittent behavior. Red arrows pointing at identified lymph nodes. (e) Quantification of intensity trend in the inflamed region over 1 min. Real-time kinetics and movements can be appreciated in Videos 3(a) and 3(b) (Video 3, MP4, 8.33 MB [URL: https://doi.org/10.1117/1.JBO.31.1.016003.s3]).

The datasets and results described in Secs. 2.1–2.5 explored the behavior of DF hypoxia signals from PpIX in the lymphatics in noninjury conditions and in a moderate wound injury model—where minimal visible inflammation was observed. The lymphatic behavior in areas of visible swelling was also studied in a mouse model where a more severe injury was inflicted, as described in Sec. 5 and summarized by representative results (N=4) in Fig. 7. Figure 7(a) depicts an example mouse model of major wound injury, where the periphery of the wound was visibly inflamed. Figure 7(b) shows the results of PF imaging of PpIX, where the recorded signal intensity (and correspondingly, measured PpIX concentration) was higher at the outside and lateral area of the wound, covering a larger area than the visibly inflamed region shown in Fig. 7(a). Hence, compared with mice with no wounds and minor wounds, PpIX concentration was higher at the wound site and the peripheral inflammation area in major injury cases. In this example, vials of oxygenated and deoxygenated PpIX are shown within the image frame as a control. Both normal and deoxygenated vials possessed a PF component, as shown in Fig. 7(b) and as previously reported for *in vitro* samples.^30^ PF frames at 1 and 13 min after the acquisition started are shown in Fig. 7(b). Figure 7(c) displays example images of the PpIX DF real-time sequence. Activity in PLN and SLN nodes was not concurrent in time. SLN nodes appeared to fluctuate across the time window, as exemplified in Fig. 7(c) for 12 and 13 min post imaging start. For severe wound models, PLN and SLN pulsations were observed on both the right and left legs of the mouse. Of note, observed pulsations did not occur at the same time for each node as indicated by the red arrows in the frames displayed in Fig. 7, indicating differences in draining activity across nodes. The dynamic behavior of the signals can be better appreciated in Videos 3(a) and 3(b). Besides observed nodes, which are highlighted with red arrows, pulsations were observed in areas located outside of the wound, close to visible swelling regions. For the representative example of Fig. 7, the intensity fluctuation of this area over time is depicted in Fig. 7(d). A total of seven pulsations were quantified for a period of 60 s or a respective frequency of ∼0.1  Hz.

Quantification of the frequency of pulsation across different time-points after 5-ALA injection in the inflammation areas and area of PLN nodes is provided in Fig. S6 in the Supplementary Material, where PLN regions pulsated at an average of 12 bpm. To obtain insight into which time points better highlight the areas of lymphatic pumping on the SLN and PLN areas as well as inflammation areas, different time points post-5ALA administration were compared in the major injury model. The results are summarized in Fig. S6 in the Supplementary Material, where 1 h post-5ALA administration resulted in a higher accumulation of PpIX and hypoxia signal in inflamed areas and in an average of 15 bpm PLN pulsations at this time point.

### 2.7 Understanding PpIX Hypoxia Signals Post-Wound Time

**Fig. 8:**
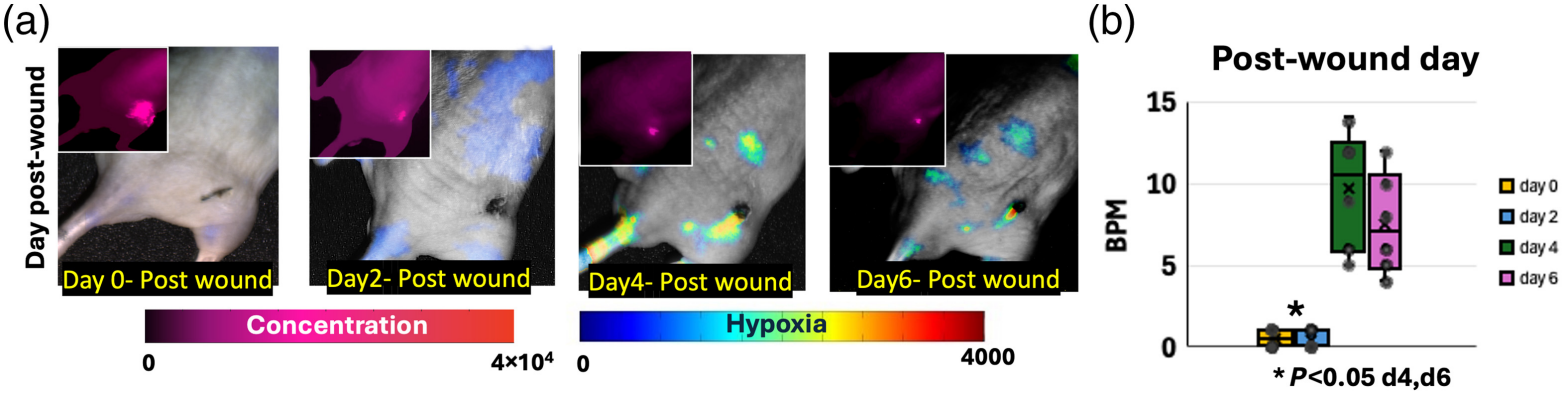
Example of lymphatic response seen in DF Hypoxia for both moderate and severe injured mouse models. (a) Hypoxia channel overlayed on a white-light image for a mouse injected and imaged every 2 days. PpIX prompt fluorescence (PF) channel representative of PpIX concentration is shown in the upper left corner for comparison. (b) Quantification of recorded pulses for the group (N=6) in beats per minute (BPM) and quantified in the SLN and PLN regions. Real-time kinetics and movements can be appreciated in Videos 4(a)–4(d) for PF of days post-wound and Videos 4(e)–4(h) for DF of days post-wound (Video 4, MP4, 8.12 MB [URL: https://doi.org/10.1117/1.JBO.31.1.016003.s4]; Video 4_2, MP4, 13 MB [URL: https://doi.org/10.1117/1.JBO.31.1.016003.s5]).

To obtain insight into lymphatic pumping and response in the SLN and PLN areas, different post-wound time-points were compared for wound models of minor injury. The results are summarized in Fig. 8. Figure 8(a) shows both prompt and DF hypoxia signals, where the 5-ALA was injected every 2 days and the mice were imaged at 1 h post-injection. PF is representative of PpIX concentration. The results showed higher PpIX PF localization and higher intensity in the wound area on all days, including day 0 post-wound. The DF hypoxia channel, as shown in Fig. 8(a) and Videos 4(c) and 4(d), resulted in pulsations that were noticeable at days 4 and 6 post-injury but not earlier on day 0 nor 2, with higher hypoxia intensity in SLN and PLN nodes on these days. Hypoxic regions and lymphatic pumping dynamics for days 4 and 6 can be fully appreciated in example Videos 4(a) and 4(b). The quantification of the recorded pulse number in BPM for a group of mice with N=4 is shown in Fig. 8(c). Pulsations at days 4 and 6 went from 4 to 12 BPM in comparison to no pulsations observed on days 0 and 2. Pulsations at SLN and PNL regions were only observed in the DF hypoxia channel and not in the PF channel. However, the PF channel displayed signals that were positioned around the wound on day 0 and localized to the smaller wound region on day 6. Given the relationship among PpIX signals, hypoxia, macrophages, and T cells, these results highlight lymphatic function and PpIX clearance being distinguishable until day 4 post-wound for wounded mouse models.

### 2.8 PpIX Lymphatic Hypoxia Response in the Presence of AsPC1 Pancreatic Tumors

**Fig. 9:**
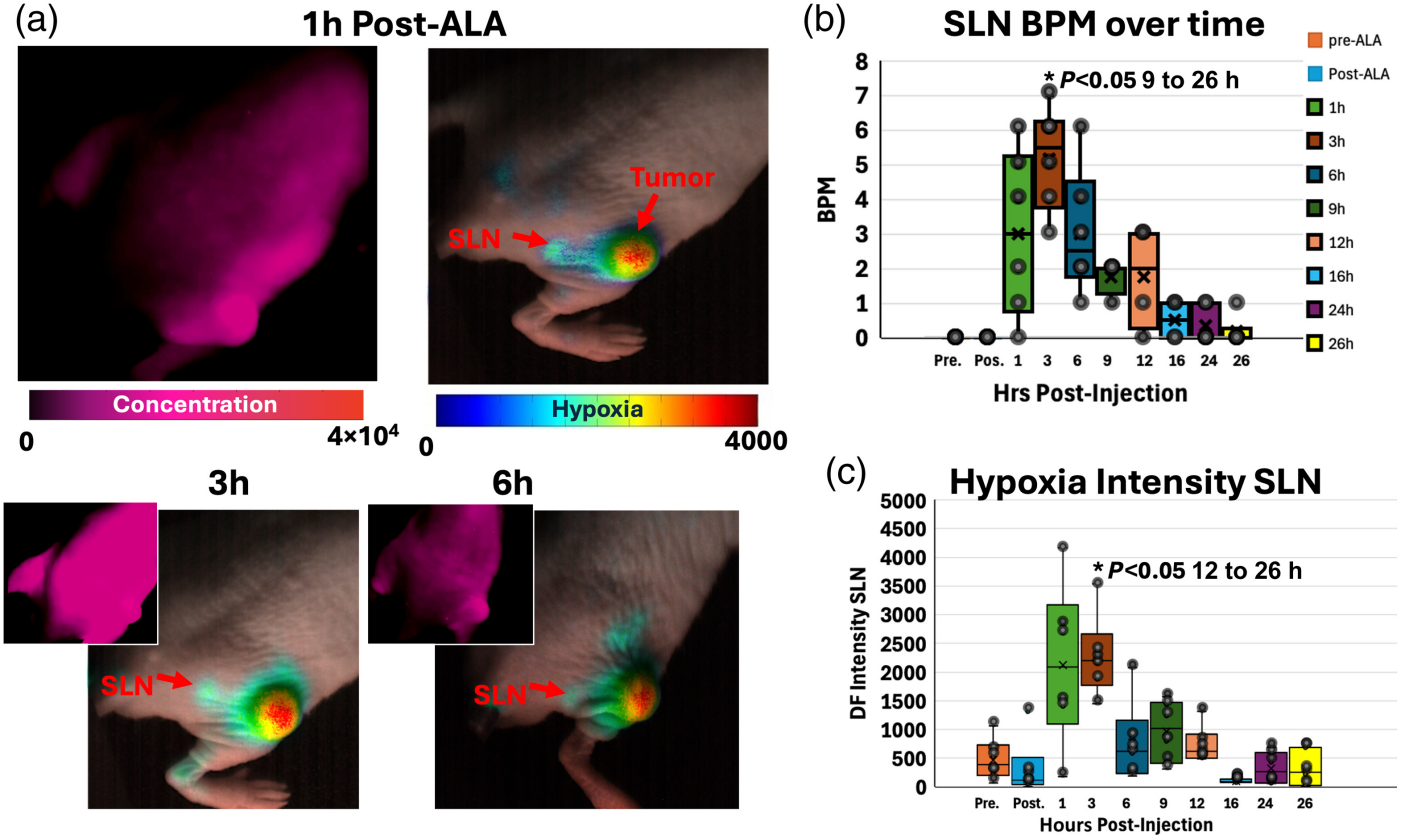
Example of lymphatic response as recorded with PpIX PF and DF imaging for a mouse with AsPC1 tumor xenograft. (a) Example sequence for time-points 1, 3, and 6 h post-5ALA injection. The hypoxia DF channel was overlaid over the white-light image for visualization purposes. PpIX PF images representative of PpIX concentration are shown in the upper left corner for comparison. (b) Quantification of recorded pulses for the group (N=6) in beats per minute (BPM) and quantified in the SLN and PLN regions across the different time points. (c) Quantification of hypoxia intensities in the SLN region per post 5-ALA time-point. Real-time kinetics and movements can be appreciated for time-points at 1, 3, 9, 12, and 24 h post 5-ALA in Videos 5(a)–5(e) for PF and Videos 5(a)–5(e) for DF hypoxia channel. P-value and * added for the group with the most statistical difference from other time-points (Video 5, MP4, 9.92 MB [URL: https://doi.org/10.1117/1.JBO.31.1.016003.s6]).

To evaluate the behavior of DF PpIX signals from lymphatics in a disease (nonwound) state, DF hypoxia lymphatic imaging was studied in mice with AsPC1 (pancreatic adenocarcinoma) tumor xenografts. The results are displayed in Fig. S7 in the Supplementary Material for imaging timepoints pre and post 5-ALA injection as well as 1, 3, 6, 9, 12, 16, 24, and 26 h post 5-ALA injection. Summary results are shown in Fig. 9(a) for 1, 3, and 6 h post 5-ALA injection. Displayed images include both PF (PF in the upper left corner) and DF hypoxia channels. DF hypoxia images have been overlaid over white-light images for visualization purposes. The pulsation rate in BPM for the SLN proximal to the tumor was quantified, and the results are plotted in Fig. 9(b). The dynamics can be fully appreciated in Videos 5(a)–5(d) for dynamics at 1, 3, 9, and 12 h post-5ALA injection in both DF and PF channels. Quantified BPM at SLN and PLN regions (highlighted) were higher at the 1 to 6 h post-5ALA injection timepoints with a higher BPM mean between 1 and 3 h post 5-ALA injection time-point. Quantification of tumor to background ratio (TBR) of PF PpIX concentration is displayed in Fig. S7(c) in the Supplementary Material, where the TBR values peak at 12 h post 5-ALA injection. DF hypoxia intensity values for the tumor region were also quantified and are shown in Fig. S7(c) in the Supplementary Material. The results displayed higher DF intensity on the tumor region at the 3 and 9 h post-injection time-points, with DF intensity decreasing on the subsequent time-points past 9 h. The DF hypoxia intensity for the SLN area was also quantified and is displayed in Fig. 9(c), where the highest hypoxia intensity was obtained at 1 and 3 h post-5ALA injection. Hence, higher hypoxia intensity in the SLN was correlated with those time points displaying higher BPM values. The results show a difference between the time points where the highest BPM and the highest hypoxia at SLN are observed (1 to 3 h) and the time points in the tumor region with the highest TBR for PF concentration (12 h) and the highest hypoxia intensity (9 h). This gives insights into the kinetics of the probe across tumors and lymph nodes being different. The overall results at peak time points display higher intensity for hypoxia in the tumor region [Fig. S7(c) in the Supplementary Material] averaging 6500 intensity counts versus hypoxia observed in the SLN regions [Fig. 9(c)] approximating 2500 intensity counts. This is expected given the size difference between the induced tumors and lymph nodes in mice.

## 3 Discussion

The experiments here demonstrated the first use of PpIX hypoxia imaging as a way to visually assess lymphatic function in real time. Part of the goal was to understand the dynamics of lymphatic function following wound injuries as well as AsPC1 tumor xenografts in mice. To our knowledge, this is the first time that lymph hypoxia has been documented *in vivo* in the whole body, and this is the first systematic study to interpret the nature of this in terms of lymphatic response to infection and cancer. Images in Fig. 2 show how lymph hypoxia appears with wound injury and how the location of hind-leg lymph nodes was confirmed by local ICG lymphatic mapping of SLN and PLNs. Although ICG lymphatic imaging can visualize local networks and flow, the hypoxia imaging can, in principle, give some functional and metabolic information about oxygen consumption.^8^ Previous studies have reported on the use of SLN and PLN nodes to drain this area in both rat^31^ and mouse models.^26^ Localization of the signal to the SLN node was also verified by opening the skin of the mouse and comparing the location of the SLN node in the DF hypoxia channel with respect to ICG, as shown in Fig. S1 in the Supplementary Material. No pulsations were observed on the ICG recordings in comparison to DF lymphatic imaging, where pulsation dynamics for SLN and PLN regions are observed, as seen in Video 1(a) for DF imaging and Video 1(b) for ICG, whereas DF imaging displayed pulsation effects, as highlighted in Fig. 2(a).

One key component of this study was to confirm that respiration and heart rate were not directly linked to the observed lymph pulsations. The average of the observed macroscopic PpIX hypoxia pulsations in lymphatic regions was ∼10 BPM. This value is ∼10× slower than the recorded respiration rates and ∼55× slower than the recorded heart rates, shown in Fig. 2(a). These lymphatic rates agree well with previously reported values where lymphatics have been observed to pump at rates from 2 to 15 BPM for mouse experiments.^32^^,^^33^ Excitation and exposure times were maintained constant throughout the experiments for a total time of 12 min per mouse. The optical power density was held steady at $5\;\mathrm{mW}/{\mathrm{cm}}^{2}$, with the field of view and distance to the imaging plane unchanged. The 5-ALA dose was consistently set at 250  mg/kg. Anesthesia parameters were standardized across all mice, with induction at 3% isoflurane and maintenance at 2% to 2.5%, using 100% oxygen at 500  mL/min as the carrier gas. No photobleaching was observed, as confirmed by PpIX control vials placed within the field of view. These controls exhibited prompt and delayed PpIX fluorescence intensities consistent with those measured *in vivo*. If photobleaching had occurred, a decrease in prompt fluorescence over the imaging period would have been expected, which was not observed in neither the mice or vials, as shown in Fig. S8 in the Supplementary Materials. Given the low irradiance used for excitation ($5\;\mathrm{mW}/cm^{2}$) and the total energy fluence ($<4\;J/cm^{2}$), reactive oxygen species generated via PpIX’s photosensitizing properties are expected to be negligible, particularly when compared with typical irradiances used for photosensitizer activation (50 to $100\;\mathrm{mW}/cm^{2}$).^34^^–^^36^ Because of this, significant PDT effects across different days were not expected; however, future studies can look at the side effects of long-term longitudinal imaging and repeated 5-ALA dose, across different tissue types, which was not herein assessed. The use of a ROS-modulating condition is also desired to understand the effect of ROS (if present) on lymphatic response. In addition, the effect of 100% oxygen as a carrier gas for anesthesia versus 100% air should be assessed to better understand changes in correlation to tissue anesthesia/oxygen levels.

The results from this study point out that lymph node pulsations are not caused by the presence of PpIX but instead only reflect the intrinsic frequency range of lymphatic fluid clearance. The results have shown that pulsations are not observed in control mice that have no injury or movement, compared with mice that have wounds and tumors or are in movement, indicating that the presence of 5ALA and PpIX is not the source of the pulsations. Therefore, it is inferred that PpIX merely serves as a contrast agent to visualize inherent pulsations that reflect changes in lymphatic clearance. The observed frequencies (0.001 to 0.2 Hz)^37^^,^^38^ overlap with those reported for vasomotion—the rhythmic contraction of micro-arteries driven by endothelial and myogenic activity. However, histological, ICG, and hypoxia imaging confirmed that pulsations correspond to lymph node regions. These regions are hypoxic and contain PpIX; hence, they are isolated in the hypoxia imaging channel but not on the prompt fluorescence channel. If vasomotion were a major driving factor of the signal, it would be expected to see oscillations throughout the mouse skin due to micro-circulation^39^^,^^40^ rather than localized regions that were histologically confirmed as lymph nodes.

Structural microscopy showed the biological structure origins of the PpIX locations, as summarized in Fig. 3, where PpIX was concentrated in a connective tissue capsule surrounding the tumor. In lymph nodes, PpIX fluorescence was mainly in the subcapsular sinus and medullary sinusoids, where immune cells such as macrophages trap pathogens. In wounds, PpIX was found primarily in hair follicles and sebaceous glands but at lower levels than in tumors and lymph nodes. These findings confirm that PpIX accumulates in different regions depending upon the tissue, and IHC was used to confirm hypoxia (HIF-1α) and immune activity (macrophages by F4/80 & T cells by CD3), shown in Fig. 4 and Figs. S2–S4 in the Supplementary Material. These images confirm higher levels of HIF-1α in lymph nodes compared with muscle tissue, which correlates with lymph nodes being visualized by the PpIX hypoxia channel. High HIF-1α levels were also observed in tumors and, to a lesser extent, in wounds. In lymph nodes, the match of PpIX fluorescent areas to those showing HIF-1α, macrophages, and T cells suggests that the latter immune cells play a role in PpIX clearance and the pulsation signals observed at the macroscopic level with DF hypoxia imaging. Previous studies have also suggested the involvement of macrophages in the signal origin of PpIX fluorescence.^41^

These results infer, as depicted in Fig. 1, that immune cells in lymph nodes, specifically macrophages and T cells in the subcapsular and medullary sinuses, aid in the clearance of excess PpIX. As lymph nodes possess low oxygen tension (∼10  mmHg), as highlighted by both IHC HIF-1α microscopy results and PdG4 oxygen probe localized to PLNs (Fig. 5), PpIX that enters lymph nodes displays a delayed fluorescence (DF) component that can be isolated through the PpIX hypoxia imager. Furthermore, the lymphatic clearance activity of PpIX through immune cells in the lymphatic system is expected to result in pulsations such as those observed in Figs. 2 and 7–9. Similarly, the IHC results for tumors showed hypoxia and immune cell presence in the connective tissue capsule. In wounds, PpIX was primarily in hair follicles and sebaceous glands, also correlating with immune cell presence. The results showed that lymphatic function measured in pulsations in BPM was observed to increase with the presence of a wound or tumor compared with intact mice. As per PF results, it was observed that PpIX preferentially accumulated in wounds, tumors, and related inflamed areas. It was then estimated that when inflammation was present, these areas contained higher PpIX concentration compared with tissues such as normal skin and the cleared PpIX from inflammation areas created better contrast for the hypoxia imager. For uninjured models, however, intensity in the lymph node areas was observed to increase with movement, as shown in Fig. 6, because mice with no wound injury and under anesthesia showed low intensities/contrast on the SLN/PLN regions, but when the same mouse was removed from anesthesia and imaged while ambulatory, intensity values in PLN and SLN increased creating better hypoxia contrast. In this case, only DF hypoxia intensity was quantified for five frames where the mouse kept a similar position—however, quantification of BPM upon movement becomes more challenging. Hence, BPM quantifications in this study were limited to when mice were under anesthesia and to PLN and SLN nodes, which are more superficial. To see the DF contrast, two conditions must be met: the existence of hypoxia and the presence of PpIX. These result in PpIX producing a delayed fluorescence component. The PF channel does not isolate hypoxic regions as it only represents the presence of PpIX and its concentration and PpIX accumulates throughout the mouse (e.g., skin), which hinders the ability to observe hypoxic areas (lymphatics) by PF alone. The reason why lymphatic pulsations were not observed through ICG imaging is still not fully understood. To better assess this, future studies should microscopically analyze if T cells and macrophages uptake ICG in a similar fashion to PpIX.

When severe injuries were imaged, areas of visible inflammation as well as their proximity displayed high DF hypoxia intensity as well as pulsation rates at ∼15 BPM. Previous studies^1^ have reported on the importance of the lymphatic system in controlling inflammation by managing the drainage of extracellular fluids, inflammatory signals, and white blood cells. During inflammation, lymphatic vessels have been reported to become significantly enlarged and more permeable.^42^^,^^43^ The expansion of the vascular network has been associated with enhanced lymphatic clearance function.^44^ This phenomenon could explain why the frequency was faster in the case of visual inflammation—however, future studies will be aimed at verifying this hypothesis.

Another important aspect is the period after the wound, where more lymphatic pumping was observed. Pulsations were discernible at the SLN and PLN regions until day 4 post-wound. This indicated that the lymphatic response was not immediate after wound formation nor distinguishable until day 4. Future work might use higher resolution macroscopic imaging to better resolve low-level PpIX hypoxia signals and vessel structures in mice. However, this occurrence can also be due to the immune response components involved in the nature of the signal. Previous studies have reported on increased dermal lymph vessels 3 days post-wound,^45^^,^^46^ as well as lymphatic dilation observed at 1-week post-wound.^47^ Additional studies have also observed a roughly twofold increase in the number of macrophages in wounds from VEGF-A transgenic mice at day 7 post-injury.^48^ These studies support our in vivo results observed at days 4 to 6 post-injury.

The use of 1 h post 5-ALA injection suggests that, even though the technique can visualize lymphatic function in real-time, the time-points after 5-ALA administration affect the output DF intensity for lymphatic regions. PpIX is eventually synthesized into heme, and previous work has reported on the clearance of the probe for both delayed and PF, with a respective decrease in intensity.^49^ DF intensity on the lymphatic SLN and PLN regions is higher at 1 and 3 h post-5ALA injection for both wounds and AsPC1 tumors. As the highest TBR contrast for PF and DF in the tumor is observed past 9 h, this gives insight that PpIX cleared by the lymphatic network is not only being cleared from the tumor area but also from other regions of the mouse. Hence, future work could look into how different 5-ALA delivery methods could enhance the signal intensity observed at the 1 to 3 h window in lymph nodes and other lymphatic structures.

The work presented here suggests that when 5-ALA is injected, promoting PpIX production, the PpIX that is produced across tissues accumulates in areas of inflammation in wounds and in the tumor. This was validated by the increase in PF intensity specific to the wound and tumor regions. It was also observed that PpIX is cleared through the lymphatic network, highlighting nodes that are actively pulsing to drain the wound or tumor areas. These nodes cannot be observed through PpIX PF imaging alone but can be seen through the DF hypoxia channel, which is representative of hypoxia, validating the notion that lymphatics are hypoxic. This hypoxia condition has been reported as an important factor for lymphangiogenesis and metastatic progression through lymph channels^16^^,^^50^; however, this is the first technique to exploit hypoxia for real-time imaging of lymphatic function.

One of the limitations of this study is the lack of a technique to directly compare lymphatic function and hypoxia in real time and *in vivo* at similar frame rates as the proposed DF imaging approach, although in some ways, this is the value of the discovery here. ICG is now used to localize lymphatic structures connected to local injection sites such as the footpad, leading to hind-foot SLN and PLN connected nodes. Hence, the quantification of lymphatic activity is limited to these areas where we could obtain ICG validation, even if DF imaging displayed additional areas of activity. Although, ideally, we might quantify the number of nodes that become active across the whole mouse, this would require further validation across modalities with previous knowledge of the location of nodes (e.g., ultrasound, ICG imaging). An additional observation was that, when covered or pressed,^49^ skin also exhibits a hypoxic response as skin also has high PpIX content at specific timepoints.^49^^,^^51^ This technique may help the understanding of the relationship between skin and lymphatic response of superficial nodes. The wound and tumor models in this study were positioned in the right hind leg of mice to control areas of drainage to the PLN and SLN nodes. It would be ideal to understand the location of the active draining lymph node and the dynamics across the mouse body with the tumor location. To keep lymphatic structures intact, this study was limited to nodes such as the PLNs and SLNs located under the skin. Even though DF hypoxia was measured on some exposed nodes, one challenge is to guarantee the integrity of lymphatic channels and nodes when opening the skin, specifically for mouse models. The current macroscopic imager does not have the required spatial resolution to visualize which lymphatic structures are driving the movement within nodes (e.g., lymphangions, node capsules, hilum) *in vivo*. Future work involves the creation of such an instrument. In future studies, the use of larger animal models might better represent the nodes and lymphatic function in humans, as well as help with the validation of imaging when skin is intact and during lymphatic surgery.

## 4 Conclusion

PpIX provides a tool for hypoxia imaging of lymph fluid and has been presented as a fundamentally new method to visualize the activity of lymph nodes and vessels under situations of nearby duress. To our knowledge, lymph hypoxia has not been characterized *in vivo* before, and this use, as a contrast for functional imaging, shows that lymphatic vessels are one of the most common naturally hypoxic systems in the body.^15^^,^^16^ Intracellular-produced PpIX (5-ALA induced) appears to be absorbed and cleared by the lymphatic network and exhibits a DF hypoxia component representative of the low oxygen conditions in the lymphatics, especially when stressed by injury or tumor growth. Hypoxia imaging can be used to selectively visualize lymphatic structures that are being used to naturally clear PpIX from inflammatory areas. The hypoxia signal is not necessarily apparent in normal anesthetized mice, presumably due to a lack of lymphatic activity, but when the animal is awake and moving, the signal can be seen in the lymph nodes of the hind legs. The localization of PpIX corresponds with areas of macrophages and T cell uptake, indicating its potential to understand immune response and function. Furthermore, at appropriate time points of 1 to 3 h post 5-ALA administration, this technology can also visualize other hypoxic regions such as tumors or areas of inflammation in wounds. The time at which hypoxia signals are higher in tumor areas is different from the time at which signals are higher in lymphatics. This highlights the potential to discriminate hypoxic signals based on time points using the same technique. Hence, this approach has the potential to uncover relationships between structures such as wounds and tumors and lymphatic clearance rates. Real-time imaging enabled *in vivo* visualization of lymphatic function and clearance of areas of inflammation, and the quantification of BPM as a metric may represent clearance rates. This imaging study was done using 5-ALA, a contrast agent commonly used in humans today. Hence, it is expected that this new contrast mechanism and imaging tool will allow for lymphatic system diseases such as lymphedema to be assessed from an oxygen content perspective, in addition to lymphatic structure and flow.

## 5 Materials and Methods

### 5.1 Mechanism of PpIX Delayed Fluorescence Imaging

The principle behind PpIX DF hypoxia imaging is explained in Fig. 1. PpIX is naturally produced by organisms in the mitochondria.^52^ PpIX synthesis is boosted by the administration of FDA-approved 5-ALA into the organism.^19^ 5-ALA is a precursor in the heme pathway. Tissues containing PpIX when excited by light (350 to 650 nm with 635 nm used in this work) emit fluorescence from 600 to 750 nm. This fluorescence can be classified into PF and DF. PF of PpIX is the most used^52^^–^^55^ as its signal originates from the excited singlet state (S1) returning to the ground state. PF is straightforward to excite through continuous wave illumination and can be collected through noncomplex cameras (e.g., cellphone cameras).^56^ PF has been effectively applied in procedures such as guided neurosurgery of glioma tumors, bacterial assessment, and PDT studies.^54^^,^^57^^,^^58^ On the other hand, DF of PpIX arises from emissions of the S1 excited state that have been replenished through reverse intersystem crossing from the triplet state (T1) (Fig. 1), where oxygen quenching takes place.^20^^,^^21^^,^^59^ Therefore, DF fluorescence appears only in the absence of oxygen or hypoxia, thereby marking regions with low oxygen content^30^^,^^59^^,^^60^ [e.g., lymphatic systems (LS), tumors, areas of inflammation] selectively visible. This is not true for normoxic tissue, where the PpIX DF signal is quickly quenched by tissue oxygen.^59^ DF has a long fluorescence lifetime (3 ms)^22^ compared with PF’s nanosecond lifetimes (<12  ns).^61^^,^^62^ Hence, the DF signal can be isolated using time-gated cameras and pulsed illumination.^49^ The use of a detector grid (e.g., CCD) allows for spatial mapping of delayed fluorescence (DF) representative of hypoxia. DF of PpIX has been recently explored as a method for real-time surgical guidance of tumors,^49^ evaluation of oxygen content in burns,^63^ and PDT assessment.^59^

### 5.2 PpIX Delayed Fluorescence Imaging Instrumentation

The pre-clinical DF hypoxia imaging setup is depicted in Fig. 1. Macroscopic imaging of PpIX DF hypoxia imaging was accomplished for a 6×6  cm field of view (FOV). The FOV was illuminated by a 635 nm pulsed LED (SOLIS-620D, Thorlabs, Newton, New Jersey, United States). To provide higher wavelength specificity, the illumination was coupled to a 635 nm band-pass filter. The illumination used a pulse width of 225  μs at a frequency of 2 kHz with a power density of $∼6\;\mathrm{mW}/{\mathrm{cm}}^{2}$. A 635 nm illumination is chosen as lower absorption and scattering from tissue chromophores are observed in this band.^64^ This would allow for penetration depth greater than 0.5 mm, which is the approximate thickness of mouse skin.^64^ To collect DF hypoxia signals, the setup employed a time-gated emICCD camera (PIMAX-4, Teledyne Princeton Instruments, Trenton, New Jersey, United States). The camera was synchronized to illumination pulses and was time-gated to capture after each illumination pulse to avoid PpIX PF and collect only PpIX DF. The camera collected at a gate width of 250  μs as previous work has shown how temporal oversampling aids to increase the collected DF signal intensity.^22^ This allows us to not need the collection of the full DF lifetime decay because, to form the DF hypoxia image, only time-gated intensity information is used. As the DF signal is amplified, visualizing features with less hypoxia/DF intensity becomes plausible. PpIX prompt fluorescence was captured during each illumination pulse. As DF is expected to be several orders of magnitude lower than PF, the signal during pulses is considered PF dominant.^20^ After each pulse, a 25  μs delay (d) was added to avoid any remaining excitation bleed through. To correct for PF and PpIX concentration changes, the collected DF emissions are divided by the respective PF.^65^ To isolate collected PpIX fluorescence, a 698/70 filter (FF01-698/70-25, IDEX H&S Semrock, West Henrietta, New York, United States) was used in the detection path. This allowed for the second major peak of PpIX to be collected in a wavelength range with low tissue chromophore absorption and scattering. This is referred to as the hypoxia or DF channel throughout this paper. A dichroic mirror was placed in the path of PpIX detection together with CMOS sensors 1 and 2 (Blackfly, FLIR, Teledyne Technologies, Thousand Oaks, California, United States) to concurrently capture both white light and PF signals synchronized to the time-stamps of the DF channel. Videos were collected at an effective frame rate of 20 fps, allowing for real-time imaging of hypoxia. Image frames were acquired with a pixel resolution of 1024 by 1024 pixels and a 2×2 binning applied before image analysis. In summary, the setup allows for concurrent imaging of white-light (RGB), PpIX PF, and DF at real-time frame rates. These data sets were saved as MP4 and AVI video files containing all frames recorded.

### 5.3 Mice Anesthesia Procedures

*In vivo* experiments were used to validate the biology using Athymic nude mice (Envigo/Inotiv, Inc., Indianapolis, Indiana, United States) *in vivo* in five different scenarios: Moderate wounds, visually inflamed wounds, mice with no wounds, mice in movement, and mice bearing AsPC1 Pancreatic Adenocarcinoma tumor xenografts. With the exception of mice in movement, mice were anesthetized (SomnoSuite Low-Flow anesthesia system, Kent Scientific, Torrington, Connecticut, United States) for wound procedures, 5-ALA injection, and imaging sessions. Anesthesia was done with a flow rate of 3% isofluorane for induction and 2% to 2.5% for maintenance, using 100% oxygen (500  mL/min) as the carrier gas (SomnoSuite Low-Flow anesthesia system, Kent Scientific).

### 5.4 Development of Wounds and AsPC1 Tumors in Mice

Mice were on an approved institutional IACUC animal care and use protocol with ID M006554-R01-A02 and were housed in a certified and approved vivarium at UW Madison. For imaging, they received a chlorophyll-free purified diet (TD.97184, Envigo RMS LLC, Indianapolis, Indiana, United States) to reduce autofluorescence from the skin surface, colon, and feces. Tumor studies were done with AsPC1 pancreatic adenocarcinoma tumor xenografts in these mice.^49^ For this, cells acquired from ATCC were cultured in RPMI 1640 media supplemented with 1% penicillin/streptomycin and 10% fetal bovine serum, growing in a 5% ${\mathrm{CO}}_{2}$ incubator at 37°C. The cells were collected and resuspended in a mixture of 50% Matrigel & 50% PBS and were injected into the flank of the right leg of athymic nude mice (6 to 8 weeks of age, Envigo) at a concentration of $1\times 10^{6}\;\text{cells}$ in 0.1 mL volume. Tumor size was monitored daily, and when a 7 to 8 mm diameter was reached (∼4 weeks after), mice were imaged. To generate mild wound models, 0.5 cm long wounds were created 1 cm above the flank of the right leg of athymic nude mice. Wounds were vertical and penetrated the dermis of the mouse. To create wounds, a magnification system was used for better control. This same process was repeated for severe wound models, with the exception that wounds were 1 cm long. In addition, mice that were visually inflamed were classified as the visual inflammation group. After incision, mice were sutured to allow for longitudinal assessment.

### 5.5 PpIX Hypoxia Imaging and Comparison to ICG-Based Localization

For fluorescence imaging, a 20% 5-ALA solution was injected retro-orbitally at a 250  mg/kg dose for all groups of mice when under full anesthesia. This dose was calculated as a human-equivalent dose using a $\mathrm{mg}/m^{2}$ conversion from humans to mice, resulting in the mouse dose that is equivalent to 20  mg/kg in a human.^66^ PpIX imaging was then performed at 1, 3, and 6 h post 5-ALA administration for a visually inflamed wounded mice group (N=4). This was done to understand the time points at which the DF intensity signal was the highest. After determination of the highest DF intensity time point, PpIX DF imaging was conducted under anesthesia at 1 h post 5-ALA injection in all cases. Groups of wounded mice (N=6) were then imaged at days 1, 3, 5, and 7 post initial wound. This was done with the goal of assessing differences in lymphatic response across multiple days post-wound. Control mice with no wounds (N=6) were also imaged and used as experimental controls, and two mice from each group were also imaged under movement. Each mouse was dynamically imaged for a span of 10 to 15 min. The location of pulsating node regions imaged through DF lymphatic imaging, specifically SLN and PLN nodes, was correlated with the location areas from ICG. After noninvasive PpIX imaging of the mice through the skin, and while the mouse was under anesthesia, indocyanine green (ICG) at a concentration of 0.1  mg/mL was injected subcutaneously into the second dorsal toe of the hindfoot, with the needle oriented rostrally.^26^ The ICG contrast was used to noninvasively confirm the location of the lymphatic vessels and nodes proximal to the right hind paw.^24^ The injection site was expected to form a small bleb before ICG was gradually absorbed into the lymphatic vessels. This process was expedited by gently massaging the mouse’s hind paw. After ICG injection, the lymph nodes under the skin were exposed by carefully dissecting and removing the skin from the center of the back of the mouse to the dorsal region of the mouse. ICG was imaged, and subsequently, PpIX DF was also imaged for the dissected mouse. ICG imaging was performed through the EleVision IR with Visionsense VS3 Iridium platform (Medtronic, Minneapolis, Minnesota, United States). The center of the FOV for the ICG imager was aligned to match the center of the FOV for the PpIX DF hypoxia imaging system.

### 5.6 Correlation of PpIX Hypoxia Signals to Respiration and Heartbeat Rates

Besides verifying the localization of pulsating nodes through ICG, pulsation patterns recorded through DF PpIX imaging were compared with patterns resulting from respiration rate and heart rate. During DF imaging of wounded mice (N=6), they were placed over an electrode-based vital sign monitor for rodents (Rodent Surgical Monitor+, Indus Instruments, Webster, Texas, United States), contact gel was placed between the four paws and electrodes, and respiration and heart (lead I, II, III) rates were measured. Pad temperature was set to 35°C. The instrument resolved the real-time heart rate and respiration rate values. Values were averaged over a 10 min period to match the DF hypoxia imaging time. The start of vital sign monitoring was timed to match the start of DF imaging. Averaged values in BPM for heart rate and BrPM for respiration rate were compared with BPM values estimated through the DF imaging measurements. For selected cases and for visualization purposes, waveforms of respiration and ECG were compared with waveforms observed from PpIX DF imaging.

### 5.7 DF Hypoxia Imaging Data Processing and Quantification

The data files acquired through the DF Imaging system included MP4 and AVI videos recorded for the duration of measurements. Image processing was performed in MATLAB. Processing included subtraction of background per frame, followed by 2×2 binning of the 1024×1024  pixel resolution, yielding frames with 512×512 resolution for quantification. In addition, to reduce computational burden on the pulse/BPM estimation and improve image quality, frames were averaged every five frames. A region selector GUI was developed to freely select areas of interest. For quantification of BPM, the DF imaging dataset, which contained lymphatic activity information, was used. In cases of correlation to ICG as the center of the FOV is aligned but not the FOV size, image registration is performed in relation to the silhouette of the mouse and the location of the wound. For wound sets, regions in the wound and, in some cases, inflammation regions, as well as SLN and PLN node regions, were selected, and the average temporal sequence was extracted over the duration of the video. Zero values were set to NaN before the average of the accounted timeframe.

Figure 3(d) displays an example sequence for 1 min. The peaks of the sequence were then calculated per minute, and the average BPM value was used. For cases where mice are in movement because fluctuations can be compromised by movement, quantification was limited to frames where the mouse position was kept approximately constant and to intensity-only values. For these frames, intensity values were averaged for a window of 20 frames for the SLN and PLN regions, and the process was repeated for control mice under anesthesia.

For the AsPC1 tumor DF datasets, regions of interest were selected at the tumor, SLN, and PLN regions, and BPM was calculated as previously mentioned. For DF intensity, estimation frames over the duration of the dataset were averaged, and the average intensity value was calculated for the regions of interest (ROIs) per mouse. The group statistics were plotted as shown in Fig. 5(d) for the groups for both SLN, PLN nodes, and tumor regions. To understand the relationship between PF and DF time points, the average PF intensity is retrieved for the ROIs as well as the skin area of the mouse, and PF TBR is calculated.

### 5.8 PpIX Microscopy Imaging of Tissue Specimens

Lymph nodes displaying pulsation activity through PpIX DF hypoxia imaging and/or nodes proximal to inflammation areas, tumors, and wound areas were resected and placed into a freezing mold with OCT freezing medium ensuring bubble removal and rapidly placed in a styrofoam container containing dry ice and a metal block; then, sections were stored in a −80°C freezer for subsequent frozen sectioning. Resulting slides were hematoxylin and eosin (H&E) stained and imaged in an Aperio AT2 Digital Pathology Slide Scanner system at 40× brightfield imaging. Tissues were frozen to preserve endogenous PpIX and image its fluorescence through a Nikon Eclipse TI microscope with an adapted 600-LP filter and 405 nm excitation wavelength. Exposure time, illumination power, and magnification of 40× were maintained constant across tissue type, and the full tissue section was scanned and stitched.

### 5.9 Immunohistochemistry and Multispectral Microscopy Imaging

For IHC lymph nodes, tumors and wound tissues were resected, placed in cassettes, and submerged in tissue fixative solution of 10% neutral buffered formalin. Tissues were then dehydrated, cleared, and embedded in paraffin for further sectioning. H&E-stained slides were obtained per tissue and imaged in the Aperio AT2 Digital Pathology Slide Scanner system at 40× brightfield imaging. Additional slides were prepared for antibody staining with primary antibodies CD3, F4/80, HIF-1α, and DAPI. For CD3, the ventana 790-4341 antibody was used with RTU dilution and Opal 620 to stain for T cells. For macrophages, ab6640 with F4/80 primary antibody (1:100 dilution) and Opal 570 was used. Finally, ab114977 for HIF-1α staining (1:100 dilution) and Opal 520 were used to stain for hypoxia factor 1 alpha. The resulting IHC-stained slides were imaged using an Olympus BX43 microscope with Nuance Multispectral Imaging System. Multispectral datasets were acquired and unmixed through appropriate spectral libraries for isolating the location of each antibody. The resulting individual images are composite images, which were compared with the localization of PpIX in the PpIX microscopy dataset.

### 5.10 PdG4 Oxyphor Oxygen Probe Use and Imaging

Oxyphor PdG4^67^^,^^68^ was used as an alternative probe to measure the ${\mathrm{pO}}_{2}$ values in lymph nodes in comparison to other tissue regions. In a similar way to ICG, the probe was injected into the right hind paw of mice, and the paw was subsequently massaged to distribute the probe to the PLN and SLN nodes in the right hind-leg region. Imaging with a 635 nm excitation laser and PIMAX 4 camera with a 700 LP filter was used to visualize the macroscopic localization of the probe through intensity values both on days 1 and 2 post hind-paw injection. Measurements were performed on day 2 for the probe to also distribute to nonlymphatic tissue (e.g., skin) for control measurements. Oxyphor measurements were performed with the OxyLED^69^ fiber probe system for average ${\mathrm{pO}}_{2}$ value estimation and with a PIMAX3-based setup to recover Oxyphor lifetime values per pixel, which were then converted into ${\mathrm{pO}}_{2}$ maps by following the Stern–Volmer equation and using the same predetermined constants as the OxyLED system.

### 5.11 Statistical Analysis

Data are presented as mean ± standard deviation in box plot format. Statistical significance between two groups was assessed using a paired, two-tailed Student’s t-test, whereas comparisons among multiple groups were evaluated through two-way ANOVA followed by Tukey’s post hoc test when applicable. All analysis was performed in MATLAB. A p-value of <0.05 was considered statistically significant. For better visualization, when multiple timepoints are compared, graphs display an asterisk (*) for the group with the most significant difference from others.

## Supplementary Material

10.1117/1.JBO.31.1.016003.s01

10.1117/1.JBO.31.1.016003.s1

10.1117/1.JBO.31.1.016003.s2

10.1117/1.JBO.31.1.016003.s3

10.1117/1.JBO.31.1.016003.s4

10.1117/1.JBO.31.1.016003.s5

10.1117/1.JBO.31.1.016003.s6

## Data Availability

All data in the paper will be made available upon reasonable request to the primary authors. MATLAB algorithms can also be made available upon reasonable request.
