# Supplementary material for: Imaging lymphatic function and inflammation response through hypoxia via endogenous biomarker
Source: J Biomed Opt. 2025 Dec 29;31(1):016003. doi: 10.1117/1.JBO.31.1.016003 (PMC12747121; doi:10.1117/1.JBO.31.1.016003)
Supplement: Supplementary file 1 [file JBO_031_016003_SD001.pdf]

Supplementary Information for

Imaging lymphatic function and inflammation response through hypoxia via endogenous biomarker

Marien I. Ochoa[1], Xu Cao[1,2], Matthew S. Reed[1,2], Eduard Matkovic[3], Weifeng Zeng[4], Samuel O. Poore[4], Brian W. Pogue[1,2]

[1] Department of Medical Physics, University of Wisconsin-Madison; Madison WI, 53705
[2] Thayer School of Engineering at Dartmouth; Hanover NH, 03755
[3] Department of Pathology, University of Wisconsin-Madison; Madison WI, 53705
[4] Department of Surgery, University of Wisconsin School of Medicine and Public Health, Madison, WI, 53705.

S1. Supplement for PpIX DF hypoxia dynamics in relation to vital signs

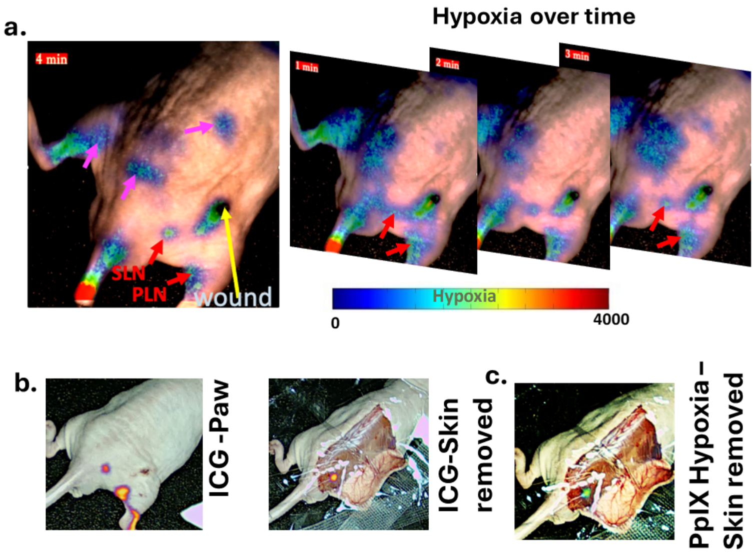

Figure S1. Example lymph response as recorded by PpIX DF imaging for a mouse with a moderate level wound injury. (a) PpIX delayed fluorescence (DF), hypoxia channel with example frames over time. PpIX DF hypoxia frame at 4 mins with lymph nodes that correlate to ICG (red arrows) and additional lymph nodes identified through PpIX DF imaging (pink arrows). (b) Intact mouse as measured with ICG and subsequently measured with skin removed (c) for both ICG and PpIX based hypoxia imaging.

S2. Supplement for Factors behind microscopic localization and hypoxia validation of tissues through IHC

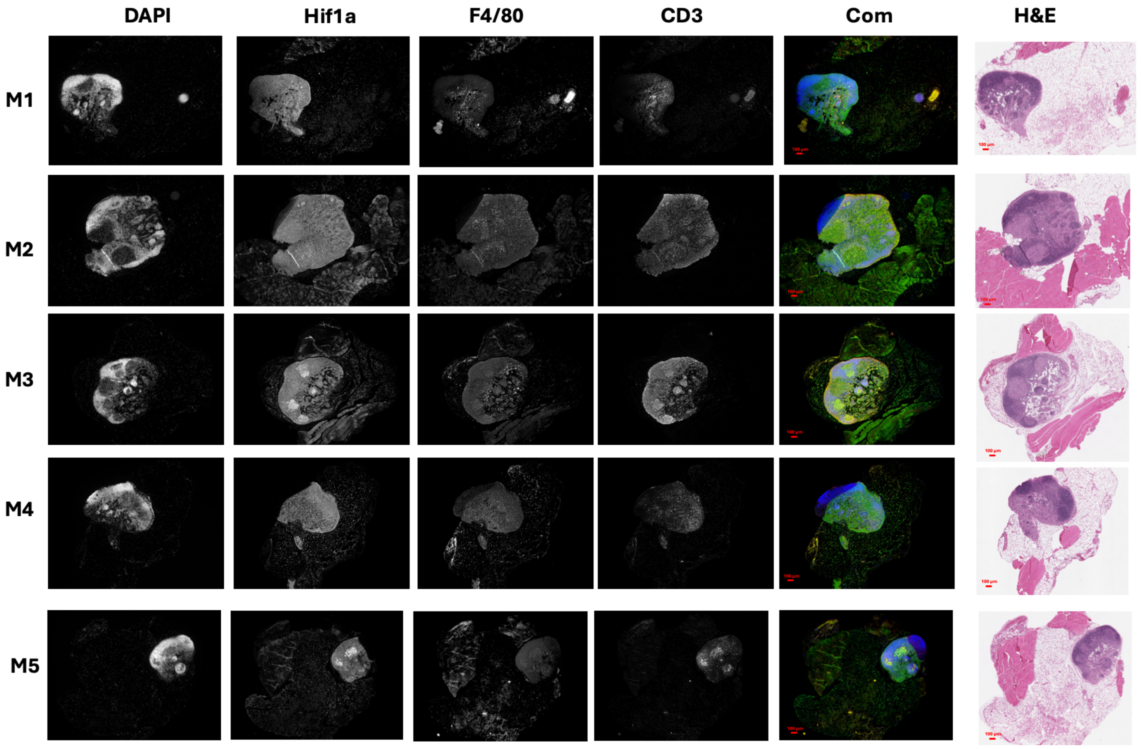

Figure S2. Verification of IHC IF in Lymph Nodes and Normal tissue at 1 hour post 5-ALA injection, with IHC staining and multispectral imaging. Example images at 40x magnification for tumor, lymph node and muscle tissue. Corresponding H&E images are provided on the last column. Additionally composite images (com) of IHC stains are displayed with individual stains displayed per column and per example mouse. Composite images are color-coded to represent DAPI in blue, HIF1α in green, F4-80 in yellow and CD3 in red.

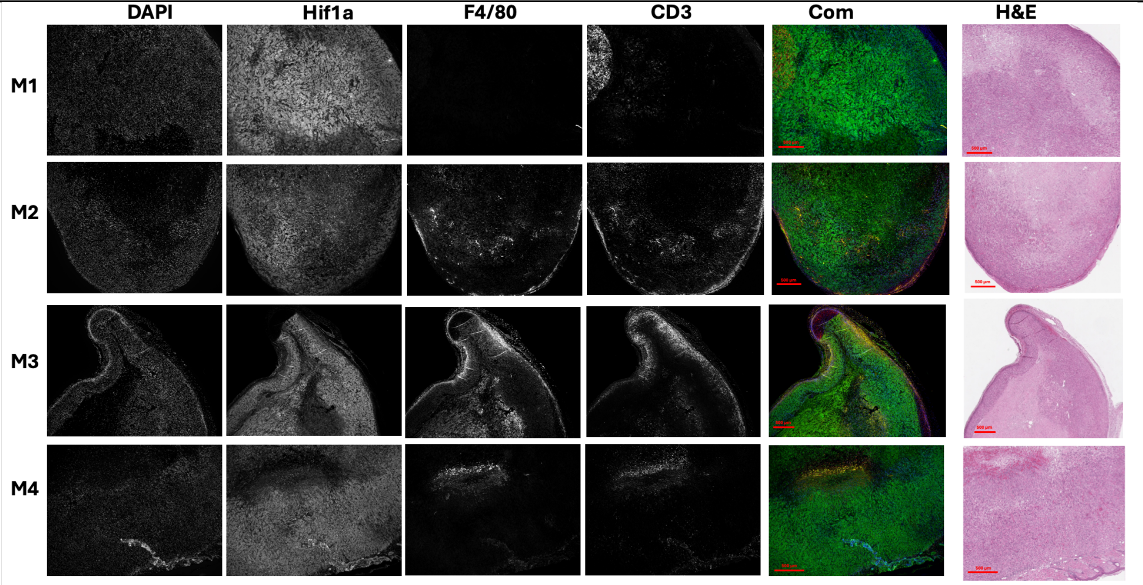

Figure S3. Verification of IHC in tumors at 1 hour post 5-ALA injection, with IHC staining and multispectral imaging. Example images at 40x magnification for tumor tissue. Corresponding H&E images are provided on the last column. Additionally composite images of IHC stains are displayed with individual stains displayed per column and per example mouse. Composite images are color-coded to represent DAPI in blue, HIF1α in green, F4-80 in yellow and CD3 in red.

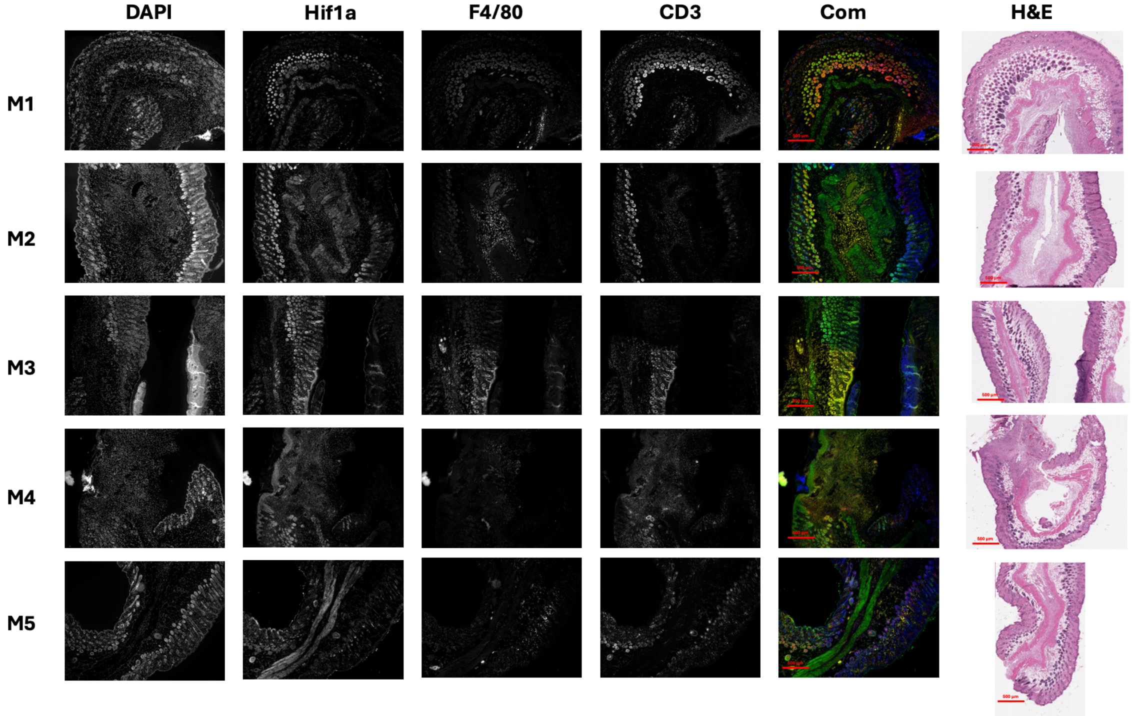

Figure S4. Verification of IHC in tumors at 1 hour post 5-ALA injection, with IHC staining and multispectral imaging. Example images at 40x magnification for wound tissue. Corresponding H&E images are provided on the last column. Additionally composite images of IHC stains are displayed with individual stains displayed per column and per example mouse. Composite images are color-coded to represent DAPI in blue, HIF1α in green, F4-80 in yellow and CD3 in red.

S5. Supplement for PpIX localization and hypoxia in vivo validation with local alternative oxygen probe.

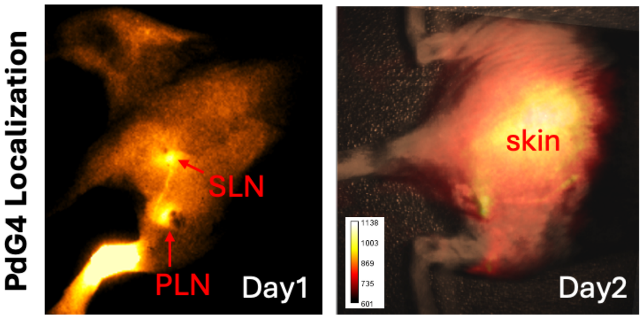

Figure S5. Images of PDG4 injectable oxygen sensing probe (Oxyphor) as localized in lymph node areas, wound areas and normal tissue. Localization of PdG4 on day 1 and day 2 post hind-paw injection.

S6. Supplement for Lymphatic response in the presence of visible swelling.

To obtain insight into which time-points better highlight areas of lymphatic pumping on the SLN and PLN areas, different time-points post-5ALA administration were compared for wound models of moderate and major injury. Results are summarized in **Figure S6.** Results for mice imaged at 1, 3,

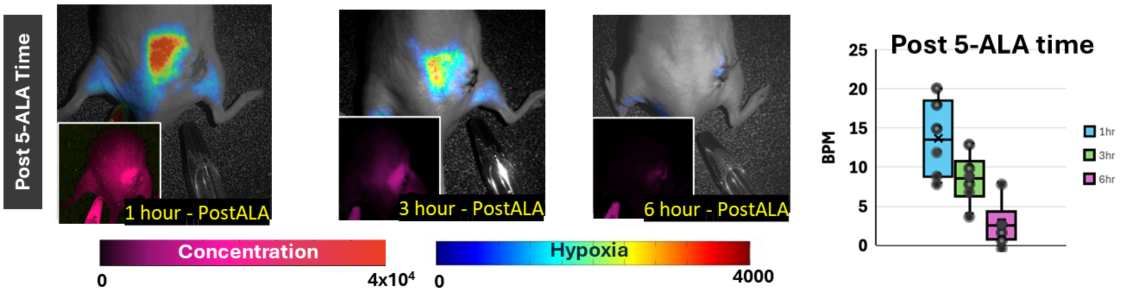

Figure S6. Hypoxia channel overlayed on white light image of a mouse with inflammation, imaged at 1, 3, and 6 hours post 5-ALA injection. PF image displayed in left bottom corner for comparison. Quantification of recorded pulses for the group (N=4) in beats per minute (BPM) and quantified in the SLN and PLN regions for post 5-ALA injection time-points. Real-time kinetics and movements can be appreciated in **videos 4(a)-(f)** for PF and DF channels for post 5-ALA injection time.

and 6 hours post 5-ALA injection are displayed in **Figure S6 (b).** Inflamed areas and regions nearby displayed pockets of DF hypoxia signal that were also visible in the PF channel. However, the SLN and PLN showed pulsations over time that were only visible in the DF hypoxia channel. This can be appreciated in **video 4 (c)** for PpIX DF hypoxia. These pulsations were not observed in the PF channel as shown in **Figure S6 (b)** bottom left corner and **video 4 (d).** The signal accumulated near areas of inflammation and displayed higher intensity and pulsation rate (BPM) at the 1 hour post-injection time-point. The intensity in areas of interest (nearby inflammation, SLN and PLN areas) for the PF and DF channels decreased from 1 to 6 hours post 5-ALA injection. Pulsations (BMP) quantified in the DF channel also decreased from 1 to 6 hours post 5-ALA injection. These are dynamically displayed in **videos 4 (c) and (d).** Quantified pulsation frequencies for SLN and PLN regions are shown in the box plot of **Figure S6** in terms of the beats per minute (BPM).

S7. Supplement for PpIX lymphatic hypoxia response in the presence of AsPC1 pancreatic tumors.

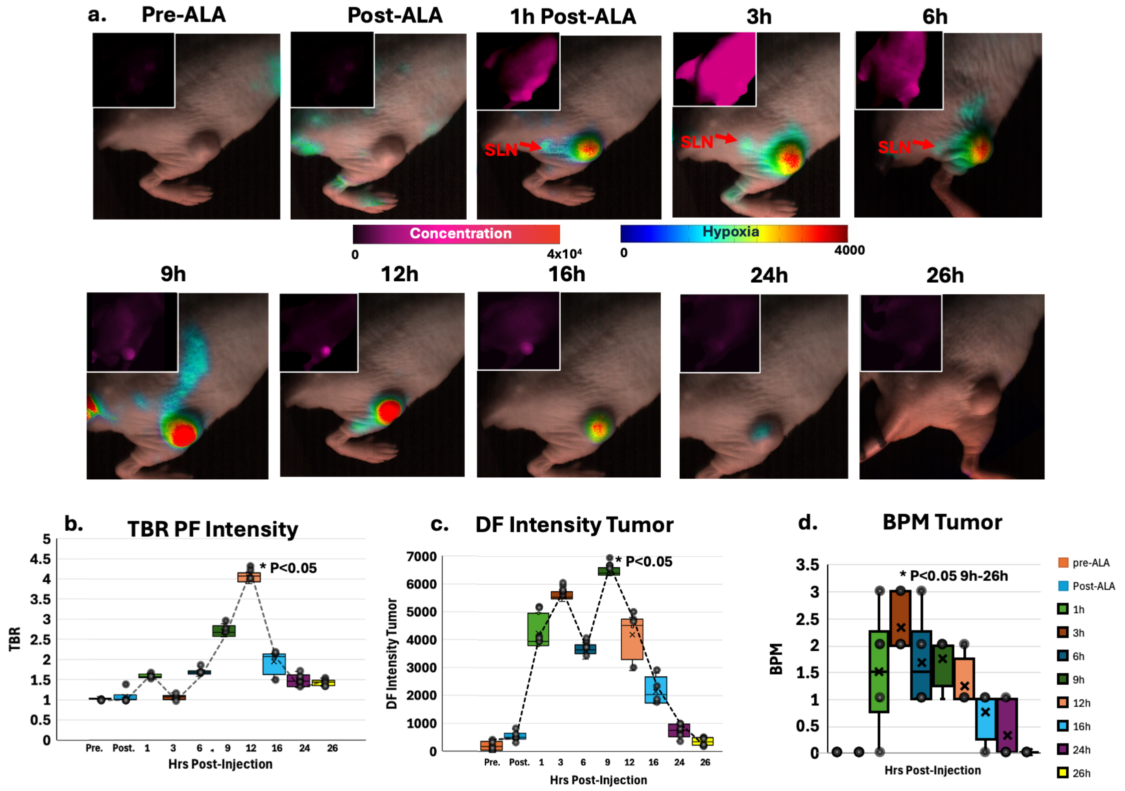

Figure S7. Example of lymphatic response as recorded with PpIX PF and DF imaging for a mouse with AsPC1 tumor xenograft. (a) Example sequence for time-points from before (pre-ALA), right after (post-ALA) and 1, 3, 6, 9, 12, 16, 24, and 26 hours post-5ALA injection. The Hypoxia DF channel was overlayed over the whitelight image for visualization purposes. PpIX prompt fluorescence (PF) channel representative of PpIX concentration is shown on the upper left corner for comparison. (b) Quantification of tumor to background ratio (TBR) per each timepoint for prompt fluorescence (PF). (c) DF intensity on the tumor area per each time-point. Real-time kinetics and movements can be appreciated for time-points at 1, 3, 9, 12, and 24 hours post 5-ALA in **videos 5(a)-(e) for PF and videos 5(f)-(j)** for DF hypoxia channel. (d) Pulsation as quantified on tumor area. Group with significant difference $P<0.05$ from all other groups indicated with *.

S8. Supplement for photobleaching during imaging

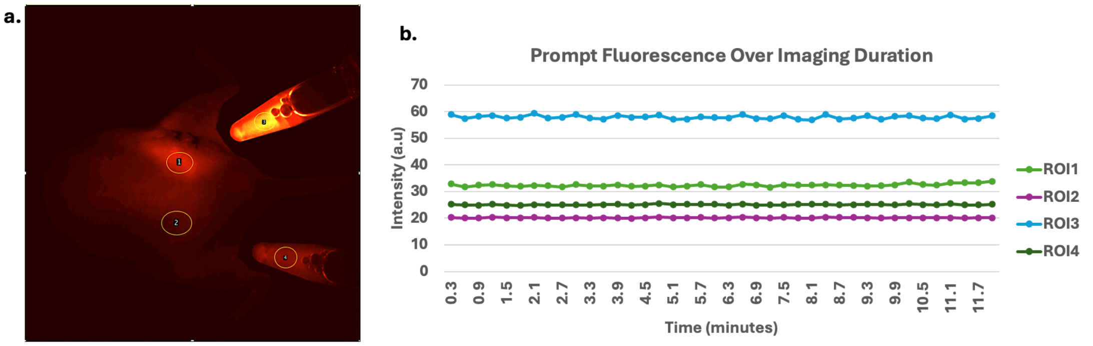

Figure S8. Example of prompt fluorescence (PF) response as recorded in vivo throughout the total imaging time and across 4 different ROIs. (a) PF intensity image averaged over time. (b) Quantification of average PF intensity per each of the ROIs over the total imaging time.

S9. Summary videos with real-time kinetics

Video 1 – Example real-time lymphatic kinetics for a mouse with a non-severe wound. (a) Hypoxia channel displaying pumping and draining kinetics from the wound to know lymph nodes. (b) ICG injected on the hind-paw of mouse to identify the two nearest lymph nodes. PLN and SLN nodes match in location with both ICG and Hypoxia imaging. (c) PpIX prompt fluorescence (PF) to demonstrate that kinetics are only observed when the hypoxia component of PpIX is isolated.

Video 2 – Example real-time lymphatic kinetics for an intact mouse with no wound. Mouse under anesthesia with low hypoxia signal in lymphatic channels. (a) Hypoxia channel displaying lymph nodes for the same mouse but now the mouse was in movement. (b) PpIX prompt fluorescence (PF) to demonstrate that kinetics are only observed when the hypoxia component of PpIX is isolated.

Video 3 – Example of real-time lymphatic kinetics for a mouse with a severe wound and inflammation region. (a) Hypoxia channel displaying high hypoxia in inflamed area and lymph nodes draining this region. The SLN and PLN which are the focus of this paper, can be observed dynamically changing in the hypoxia channel. Inflamed area also dynamically changing. (b) PpIX prompt fluorescence (PF) to demonstrate that kinetics are only observed when the hypoxia component of PpIX is isolated. Deoxygenated and normal PpIX vials were prepared and used as controls. Only the deoxygenated vial can be observed in hypoxia channel while both vials are observed in the PF channel.

Video 4 – Example of real-time lymphatic kinetics for a mouse with a non-severe wound as imaged on different days post-wound. (a) to (d) PpIX prompt fluorescence (PF) to demonstrate that kinetics are only observed when the hypoxia component of PpIX is isolated. Imaging on Day 1,3,5 and 7 post-wound. PpIX PF displays highest intensity at the wound area only. (e) to (h) display hypoxia channel kinetics for days 1,3, 5 and 7 post wound, exemplifying highest activity in sentinel lymph node close to the wound at day 5 and day 7 post wound.

Video 4_2 – Example of real-time lymphatic kinetics for a mouse with a severe wound as imaged on different hours post 5ALA admininstration. (a) to (c) PpIX prompt fluorescence (PF) to

demonstrate that kinetics are only observed when the hypoxia component of PpIX is isolated. Imaging at 1,3 and 6 hours post-5ALA administration. PpIX PF displays highest intensity at the wound area only. (d) to (f) display hypoxia channel kinetics for 1,3, and 6 hours post 5ALA administration, exemplifying highest visualization of hypoxia at 1h post 5-ALA administration possibly due to the clearance of PpIX over time.

Video 5 – Example of real-time lymphatic kinetics for a mouse with an AsPC1 tumor imaged on different hours post 5ALA admininstration. (a) to (e) PpIX prompt fluorescence (PF) to demonstrate that kinetics are only observed when the hypoxia component of PpIX is isolated. Imaging at 1,3,9,12 and 24 hours post-5ALA administration. (f) to (j) display hypoxia channel kinetics for 1,3, 9, 12 and 24 hours post-5ALA administration, exemplifying highest visualization of hypoxia in sentinel lymph node at 1h timepoint.
